# Hybrid Chemo-Enzymatic Heterogeneous Catalyst for Green and Enantioselective Hydroxylation

**DOI:** 10.1021/acscentsci.6c00674

**Published:** 2026-08-27

**Authors:** Marty Kinnaer, Justin Ovaert, Valentine Simar, Zuzana Hlavenková, David Cannella, Damien P. Debecker

**Affiliations:** † Institute of Condensed Matter and Nanosciences (IMCN), Université catholique de Louvain (UCLouvain), Place Louis Pasteur, 1, 1348 Louvain-la-Neuve, Belgium; ‡ PhotoBioCatalysis−unitUniversité libre de Bruxelles, 165/61 - Avenue F.D. Roosevelt, 1050 Brussels, Belgium; § Central European Institute of Technology, 37748 Masaryk University, Žerotínovo nám. 617/9, 601 77 Brno, Czech Republic

## Abstract

The implementation of green chemistry principles in catalysis is essential for sustainable production of specialty chemicals, where selectivity and efficiency are paramount. Unspecific peroxygenases (UPOs) have emerged as promising biocatalysts for enantioselective C–H oxyfunctionalization reactions, using hydrogen peroxide (H_2_O_2_) as the sole oxidant. However, the instability of UPOs toward H_2_O_2_ and the nonsustainable production of this reagent remain key challenges. Herein, we report the design of a hybrid chemo-enzymatic heterogeneous catalyst integrating an Au–Pd-based material with the PaDa-I UPO mutant, using an “enzyme-in-a-cage” immobilization strategy. This bifunctional system enables controlled *in situ* H_2_O_2_ generation directly from H_2_ and O_2_, coupled with the enantioselective hydroxylation of ethylbenzene in a single reactor. The catalyst exhibits high activity, excellent enantioselectivity (ee = 97.7%), and stability without enzyme leaching. This approach offers a robust and sustainable platform for one-pot chemo-enzymatic cascade reactions in chemical synthesis.

## Graphical abstract

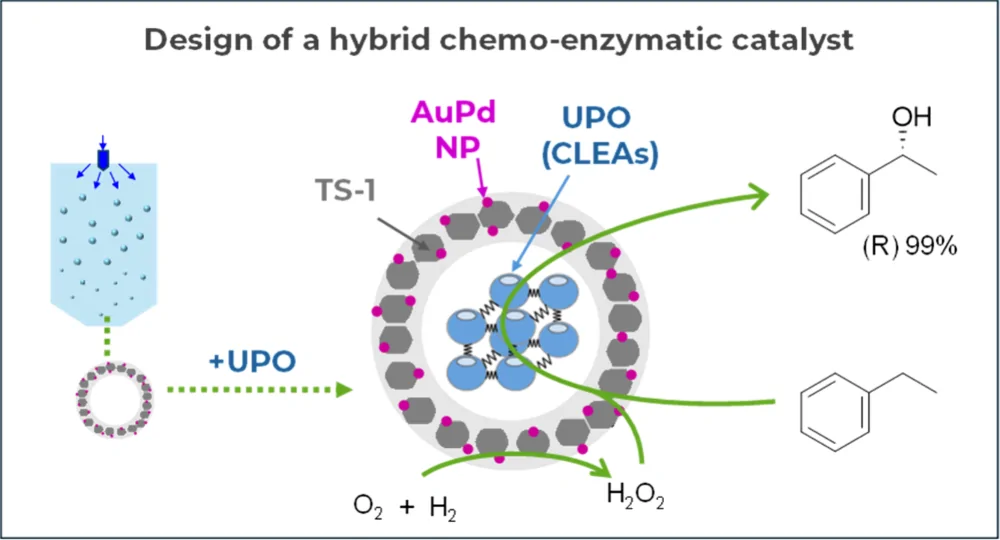

## Introduction

The integration of green chemistry principles in chemical processes is crucial for advancing sustainable chemical manufacturing.^1^ In the field of catalysis, atom economy, waste prevention, and energy conservation are arguably the most important principles of green chemistry. This is especially true when it comes to specialty chemicals, where added value often surpasses the economic interest of reducing waste materials and energy.^2^ Biocatalysis has risen as one of the main actors in the production of specialty chemicals. The high selectivity of enzymes, along with their mild reaction conditions and low toxicity, make them an obvious choice for greener chemistry.^3,4^

Unspecific peroxygenases (UPOs) are increasingly attractive in the field of biocatalysis thanks to their ability to catalyze the oxyfunctionalization of C–H bonds.^5^ It is now well established that UPOs can accept a wide scope of substrates and still display a near perfect regio-and-stereoselectivity.^5–8^ Unlike cytochrome P450, the original oxyfunctionalization biocatalyst, UPOs require no organic cofactor such as NAD­(P)H and rely solely on H_2_O_2_.^5,9–11^ In the past decade, UPOs have been increasingly studied and have shown high potential for directed evolution. Researchers have thus created mutants with improved resistance to organic solvents, or with various predefined selectivities.^9,12–15^ This would make them a great platform to explore industrially relevant reactions to produce pharmaceutical intermediates^16,17^ and specialty materials (catalysts, sensors, polymers, ...),^18–20^ where chirality is key.

These advantages, however, come at a cost. Hydrogen peroxide, needed as an electron donor for the UPOs, bears the advantages of being much cheaper than nicotinamide cofactors needed by the P450 enzymes, for example. But as a strong oxidizing agent it can also oxidize the enzymes, causing them irreversible structural damages and ultimately inactivation.^21^ Thus, H_2_O_2_ concentration must remain low (typically in the mM range)^22^ in the medium. To reach high product concentration, H_2_O_2_ can be continuously supplied in low concentrations. This can easily be achieved using a fed-batch type of system.^23^ However, this external H_2_O_2_ is typically produced through heavy (non green) industrial processes^5,24–26^ and its use represents important EHS hazards: risks of explosions, fire and danger from human exposure (see detailed discussion on these aspects in the Supporting Information, Section S6).^27–29^

Ideally, H_2_O_2_ should be produced directly in the medium^5,25,26^ as to avoid the energy demand and waste production related to the production, concentration, transport, and storage of high-grade hydrogen peroxide. In a thorough review of H_2_O_2_-driven biocatalysis, Burek et al. laid out several enzyme-compatible H_2_O_2_ production methods.^30^ Several enzymatic cascades allow for a channeled electron transfer from a sacrificial electron donor down to the final peroxide-driven enzyme using, for example, glucose oxidase (GOX) or formate oxidase (FOX).^30^ However, such systems require a sacrificial electron donor, some of which decompose into undesirable or inhibitory products that will negatively impact the atom economy and robustness of the system. Alternatively, nonenzymatic approaches use heterogeneous catalysts to produce H_2_O_2_ through electro/photocatalysis. Notable examples include the electrochemical reduction of O_2_ ^31^ or the photocatalytic oxidation of H_2_O.^32–34^ These methods, however, have shown limited reactivity and selectivity due to the formation of radical species, damaging the enzyme and oxidizing the products.^33^

Another approach, softer to enzymes, is the combination of H_2_ and O_2_ gases which can be achieved in mild conditions and in water.^35–37^ The production of H_2_O_2_ from H_2_ and O_2_ gas using supported Au–Pd nanoparticles has been investigated for the past 20 years.^35–41^ The preparation of this catalytic material has been thoroughly improved over the years in hopes of reaching industrially relevant H_2_O_2_ concentration. These attempts have been generally unsuccessful due to the catalysts’ high activity toward H_2_O_2_ degradation.^42^ In a well-designed cascade reaction, however, the catalytic decomposition of H_2_O_2_ is not an issue, as it is meant to be consumed in the subsequent step of the reaction. Thus, AuPd-based catalysts have been showcased in several cascade reaction processes.^26,43,44^ Of specific interest, an AuPd/TiO_2_ catalyst was successfully combined with an UPO to drive the one-pot hydroxylation of ethylbenzene.^25^ In this example, the heterogeneous catalyst is recoverable and reusable, but the enzyme, used in its free form, is lost in the process. Immobilizing UPO on the catalyst would make this system even more advantageous.

Hybrid chemoenzymatic heterogeneous catalysts (HCEHC) are an emerging class of catalysts that combine biocatalysis and inorganic catalysis in a single solid.^45^ They can perform two or more consecutive reactions simultaneously in a single reactor.^45–50^ This ability to enable one-pot cascade reactions, particularly for high-value applications, makes HCEHCs promising tools for green chemistry. Their use can significantly intensify processes by merging multiple reactors into one, eliminating the need for intermediate recovery and thus reducing both costs and waste. More generally, and in the case of unstable intermediates, cascade reactions may also improve atom economy by preventing product decay through immediate consumption.^45,51^ However, many catalytic materials are typically unsuitable for enzyme immobilization strategies, so the design of these hybrid catalytic materials remains a challenge. In previous works, we demonstrated the possibility of combining a zeolite with one or more enzymes in a single solid, without loss of functionality.^52,53^ Recently, other groups have shown the possibility of creating HCEHCs with UPOs by using a similar immobilization technique. They have combined UPOs with meso- and macroporous Pd/TiO_2_ photocatalysts^54^ or Rh-doped covalent organic frameworks (COFs)^55^ to drive the production of H_2_O_2_ *in situ*. However, these methods require the use of a sacrificial electron donor, which negatively impacts the atom economy of the process. Another approach (which advantageously does not rely on noble metals) is to combine the photocatalytic production of H_2_O_2_ with its biocatalytic use;^56^ this was shown with horseradish peroxidase and should be applicable to UPO.

In the present study, we show that it is possible to combine a AuPd-based catalyst and a UPO in a single bifunctional solid material. Through an “enzyme-in-a-cage” strategy (Figure 1), we manage to immobilize the commercially available PaDa-I mutant of AaeUPO into hollow supra-particles made from TS-1 zeolite nanocrystals decorated with Au–Pd NP. The obtained hybrid catalyst effectively catalyzes the *in situ* production of H_2_O_2_ from H_2_ and O_2_ molecular gases and the enantioselective hydroxylation of ethylbenzene, in one pot.

**Figure 1:**
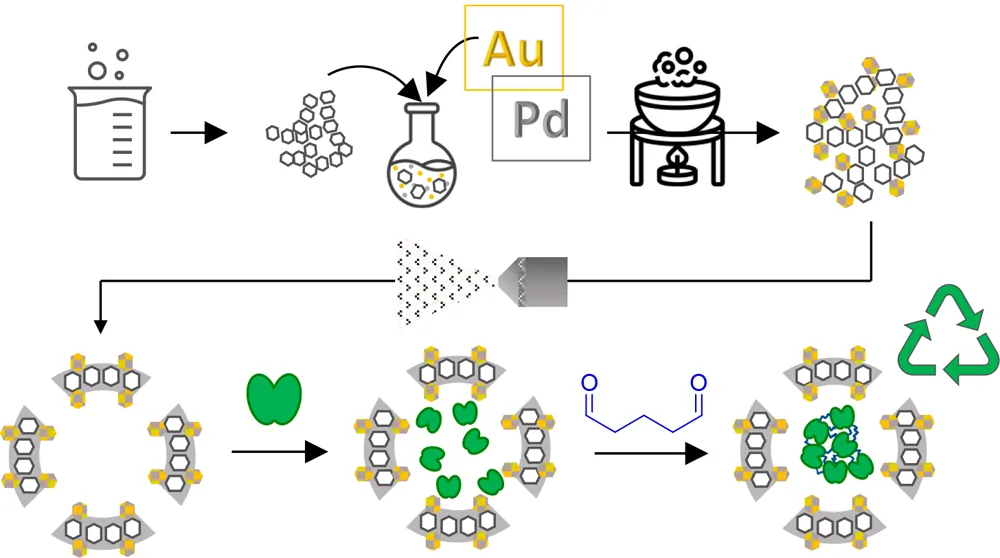
Schematic view of the catalyst synthesis process. In order: hydrothermal synthesis of TS-1 nanocrystals, wet impregnation of AuPd NP, shaping of the AuPd/TS-1 in hollow microspheres, enzyme addition, enzyme entrapment via precipitation and cross-linking.

## Results and Discussion

### Study of the Inorganic Catalyst (SD)-AuPd/TS-1

The inorganic catalyst was prepared through a multistep process (see Experimentals Section). Briefly, TS-1 zeolite nanocrystals were prepared^52^ and then impregnated with Au and Pd (0.5% w/w each) nanoparticles (NP) following a procedure well-known from the literature.^57^ Then, this pristine AuPd-decorated zeolite was suspended in water, supplemented with tetraethylorthosilicate (TEOS), and spray-dried to form stabilized hollow microspheres.^58^ X-ray photoelectron spectroscopy (XPS) confirmed the presence of metallic Au and Pd, as well as the absence of Cl from the precursors (see Supporting Information, Figure S1). The actual metal loading of SD-AuPd/TS-1 measured via ICP analysis was 0.4% of each metal, which is coherent with the expected 0.5%, after addition of silica through the spray drying (SD) process. The morphology of the microspheres was confirmed by scanning electron microscopy (SEM) (Figure 2C,E). Spheres of various sizes are visible, most ranging from 1 to 11 μm (size distribution available in Figure S6). Both the TS-1 nanocrystals and the Au–Pd NPs are discernible at the surface of the spheres, as well as the silica binder. HAADF-STEM was used to accurately differentiate the metal NPs from the support (Figure 2A,B). Through EDS imaging, the metals appear to be randomly alloyed within each nanoparticle, which is typical of supported Au–Pd nanoparticles calcined above 400 °C.^41^ The size of these particles follows a single normal distribution centered around 19 nm in diameter (SD: 4 nm, *N* = 443), with some outliers of larger size. Nanoparticle sizes were determined using NanoDetect v2.2.^59^ Additional characterizations of the inorganic catalysts are available as Supporting Information (section S1: N_2_ physisorption, Hg intrusion-extrusion, additional STEM, SEM).

**Figure 2:**
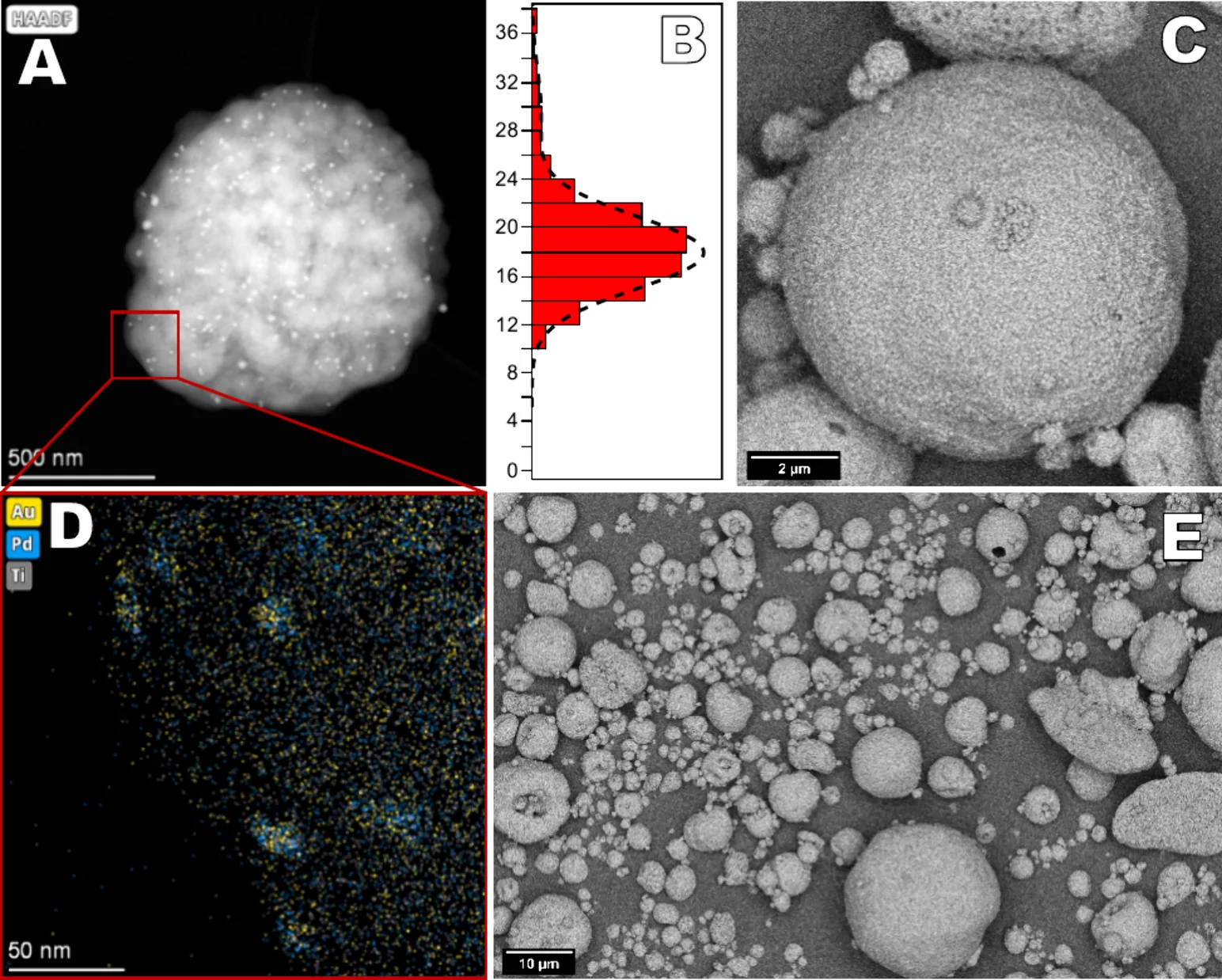
A: HAADF-STEM image of SD-AuPd/TS-1. B: metal NP size distribution from STEM images. C,E: SEM images of SD-AuPd/TS-1 microspheres. D: EDS data for Au, Pd, Ti from image A.

The activity of the inorganic catalyst (both in the pristine AuPd/TS-1 form and after shaping via spray drying, SD-AuPd/TS-1) was tested in aqueous solution as well as in an acetone/water (2/3 v/v) mixture (Figure 3A). In both cases, the water was buffered by Tris-HCl 50 mM at pH 7.6. The reactor was pressurized with a mixture of 0.3 bar of H_2_ and 2.7 bar of dry air. The measured concentrations of H_2_O_2_ are shown in Figure 3A; the production increases with the addition of acetone to the medium as well as after shaping the catalyst. Under these low-pressure conditions, it is apparent that the activity toward H_2_O_2_ is limited by gas diffusion: the intrinsic specific activity of the catalyst is a nonlimiting factor in this system. Figure 3C demonstrates that the stirring speed directly dictates the concentration of H_2_O_2_ in the medium after 1 h of reaction. Similarly, in Figure S7, it is shown that decreasing the reaction volume increased the final concentration of H_2_O_2_, while the catalyst loading had no impact on productivity. It is therefore possible to control the rate of peroxide production by controlling the stirring speed and the liquid volume. Working in the presence of acetone as a cosolvent also allows us to reach higher H_2_O_2_ productivity. The addition of acetone increases the solubility of H_2_ and O_2_ gases,^60,61^ which can enhance their transfer rate and thus catalytic activity.

**Figure 3:**
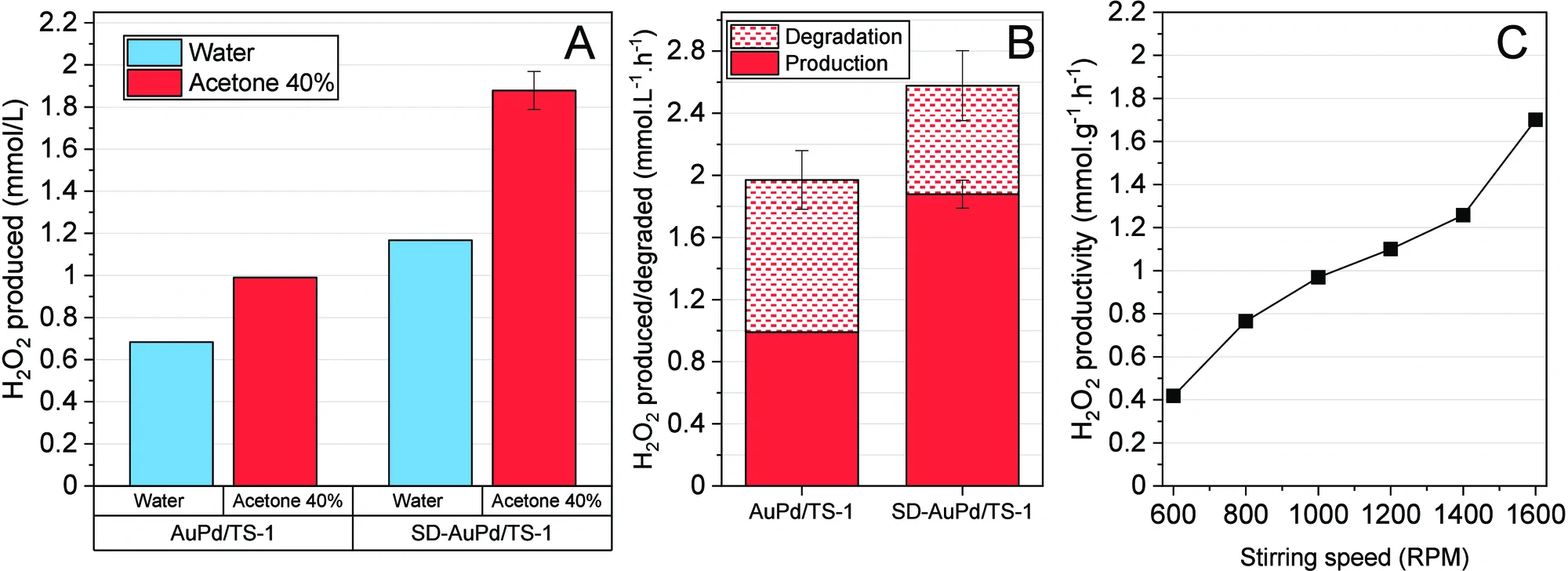
Activity of the inorganic catalysts (1 mg/mL) in water at pH 7.6 under 0.3 bar H_2_, 2.7 bar dry air. A) H_2_O_2_ concentration after 1 h in the presence or absence of acetone (40% v/v), using the benchmark catalyst (AuPd/TS-1) or the catalyst shaped by spray-drying (SD-AuPd/TS-1). B) Comparison of the production and degradation rates of H_2_O_2_ by AuPd/TS-1 and SD-AuPd/TS-1. Degradation was assessed with an initial H_2_O_2_ concentration of 7 mM. C) H_2_O_2_ productivity by SD-AuPd/TS-1 at increasing stirring rate in the presence of acetone (40% v/v).

The higher net H_2_O_2_ productivity measured for the spray-dried as compared to the pristine catalyst (Figure 3A) is tentatively attributed to a difference in the competing H_2_O_2_ degradation rate. Figure 3B shows that after spray-drying, the H_2_O_2_ degradation activity of the catalyst is lowered by 50%. This phenomenon can be explained by a difference in metal NP structure. Indeed, the size, structure and composition of the NP—all of which can be influenced by calcination temperature—are the major factors influencing the activity of Au–Pd catalysts toward H_2_O_2_ production and degradation.^41,42,62^ In this case, both catalysts are activated at 400 °C under H_2_, but SD-AuPd/TS-1 is also calcined at 550 °C after spray-drying. While AuPd/TS-1 displays small NP, below 10 nm in diameter, many of them are pure Pd (Figure S2), which are known to promote H_2_O_2_ degradation.^62^ After calcination at 550 °C, the metal NP on SD-AuPd/TS-1 grow to an average of 18 nm, with better metal dispersion and no visible pure Pd particles, which is typical of samples calcined above 400 °C.^41^ Though larger particles are not typically preferred,^42^ their better metal alloying likely increases the overall yield of H_2_O_2_.^63^

### Study of UPO and the Cross-Linked Enzyme Aggregates (CLEAs) Formation

In our strategy based on the encapsulation of the enzyme in hollow zeolite microspheres, proper CLEA formation is capital to avoid enzyme leaching. At the same time, enzymatic activity should be preserved during the process of cross-linking. Here, different CLEAs were prepared using various precipitating agents and with the enzyme either in the form of crude extract (PaDa-I) or of purified, His-tagged mutant (H6-PaDa-I). After washing with deionized water, the CLEAs were tested for the hydroxylation of ethylbenzene (EB) to 1-phenylethanol (PhEtOH) in the presence of 1 mM of exogenous H_2_O_2_.

All CLEAs show significant activity toward PhEtOH (Figure 4) and reach final concentrations close to that reached by the free enzymes. Only ethyl lactate showed a major negative impact on the activity of the H6-PaDa-I enzyme. All cross-linked enzymes appear to consume more H_2_O_2_ than required to produce PhEtOH as the sum of H_2_O_2_ and PhEtOH does not reach the initial 1 mM added to the medium. This phenomenon is not observed with free, pristine enzymes where no significant loss of H_2_O_2_ is observed (Figure 4, Control). As this loss was also observed in the absence of active enzyme (Figure 4, Water), it could be assumed that this hydrogen peroxide may have reacted with remaining GAH and/or the Schiff bases forming the cross-links. If this is the case, it may impact the long-term operation stability of the biocatalyst.

**Figure 4:**
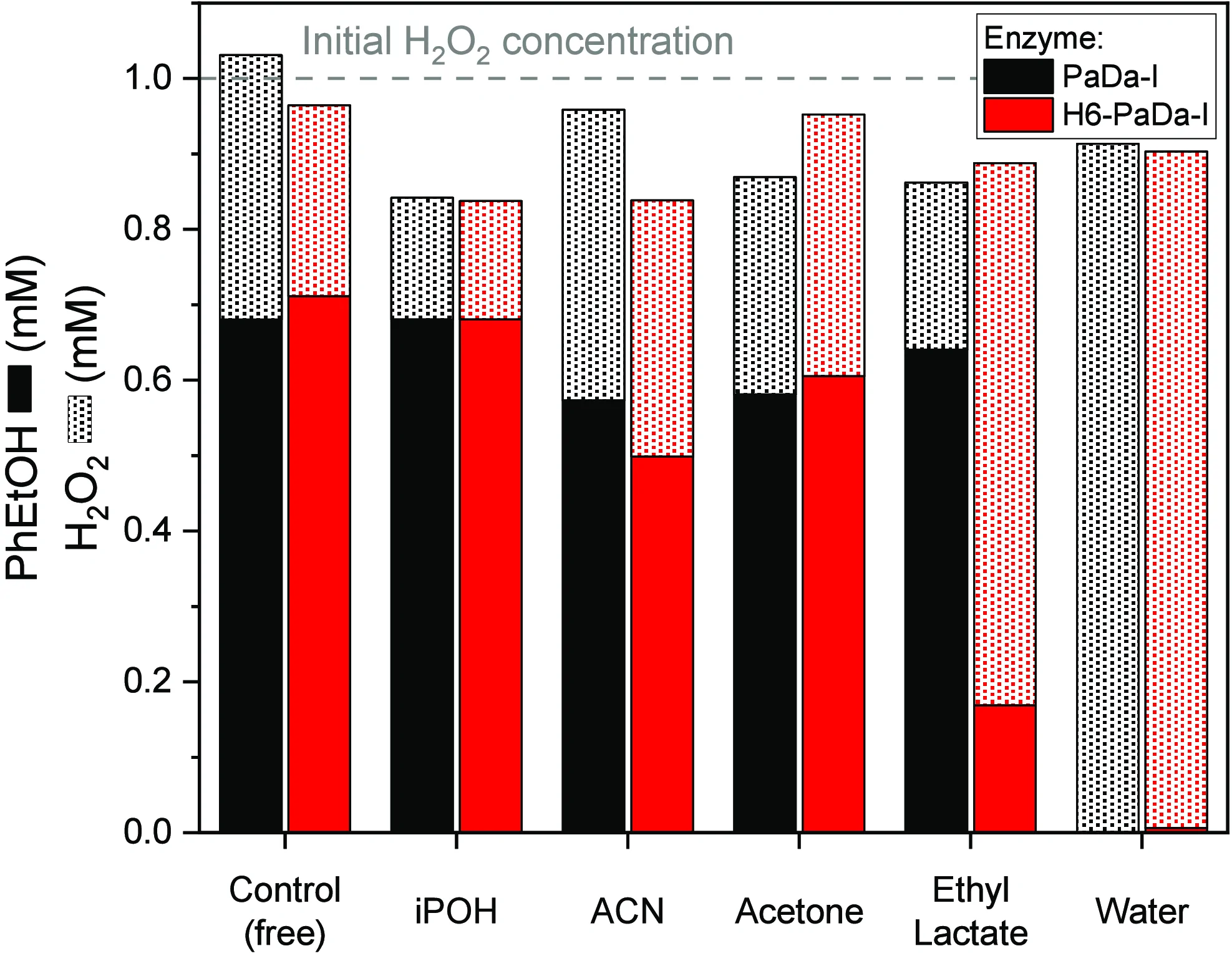
Activity of CLEAs prepared using varying precipitating agents and either PaDa-I (black) or H6-PaDa-I (red). Reaction is carried out in the presence of 1 mM H_2_O_2_ and 20 mM EB. The solid bar represents the amount of PhEtOH produced. Shaded area represents remaining H_2_O_2_. Positive control experiment (left) represents the activity of the free enzyme. Negative control experiment (right, Water) shows the activity measured when no precipitating agent is used and no CLEAs appear to be recovered.

Considering these results, iPOH and ethyl lactate were selected to produce hybrid catalysts. iPOH was chosen for yielding the highest activity overall, which is coherent with a previous literature report.^64^ The choice of ethyl lactate is motivated by previous experience in our group^52^ and for its surprisingly different results between the H6-PaDa-I and native PaDa-I. Further discussion on CLEA formation is available in Section S3.

### Study of the Cascade Reaction with Free UPO and SD-AuPd/TS-1 Catalyst

In accordance with a previous report in the literature,^25^ combining the AuPd-based catalyst (here SD-AuPd/TS-1) with free PaDa-1 showed that the enzyme can convert EB to PhEtOH with very limited overoxidation to acetophenone (Figure 5A) with no external source of H_2_O_2_. Furthermore, the production of PhEtOH (2.6 mmol g^–1^ h^–1^; 38 000 mol_product_ mol_enz_^–1^ h^–1^) appears faster than the measured production of H_2_O_2_ (1.8 mmol g^–1^ h^–1^) by the inorganic catalyst in the absence of enzyme (Figure 5B). This difference is tentatively attributed to a proximity effect between the two catalysts; the immediate consumption of H_2_O_2_ prevents its subsequent degradation by the metal catalyst. Indeed, hydrogenation of H_2_O_2_ to water by the Au–Pd catalyst is known to limit the apparent H_2_O_2_ production and to increase H_2_ consumption, making the process less efficient.^35,42,62^ Consistently, the measured degradation activity of the inorganic catalyst was estimated at around 0.7 mmol g^–1^ h^–1^ in a 7 mM H_2_O_2_ solution, which is roughly the difference between net H_2_O_2_ production and PhEtOH production (Figure 5B). This experiment highlights the interest in combining the two catalysts in a single system. The stability of this cascade system contrasts with the demonstrated lack of stability when using a fed-batch approach (Figure S18).

**Figure 5:**
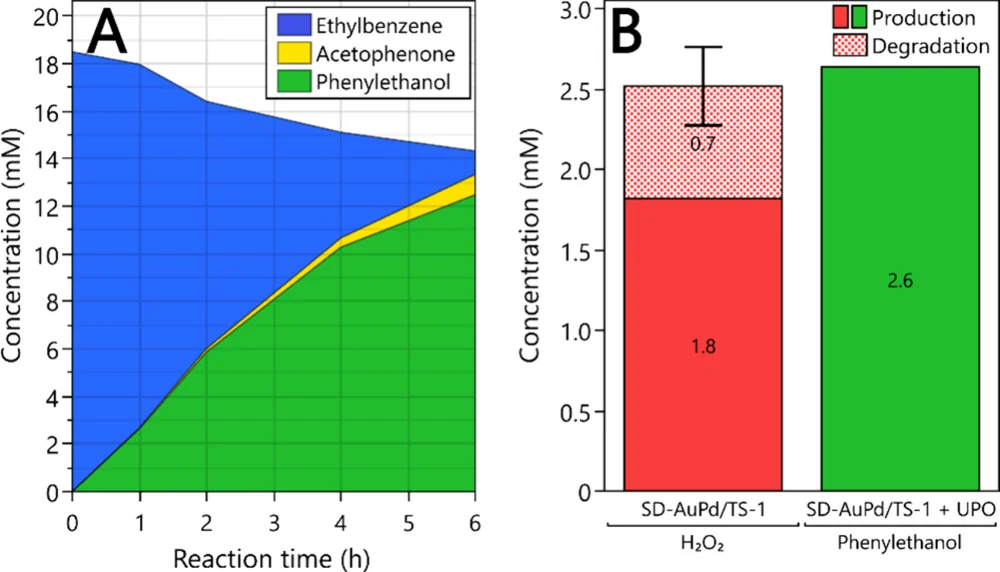
A: Production of PhEtOH from EB by free PaDa-I (3.6 μg/mL) in cascade conditions with 1 mg/mL SD-AuPd/TS-1. B: Comparison of H_2_O_2_ production and degradation by the inorganic catalyst and the production of PhEtOH in cascade conditions. The error bar is the propagated error from production and degradation standard errors (*N* = 2 + 2).

### Study of the Hybrid Catalyst

Detailed insights into the optimal synthesis parameters and operational parameters of the HCEHC are available in the Supporting Information, section S4. Briefly, it was found that the best compromise between activity and reusability of this material was achieved by precipitating the nonpurified, native enzyme solution (PaDa-I) with ethyl lactate. Reducing Schiff bases with NaBH_4_ was found to decrease activity without sufficient gain in CLEA stability. To limit the risk of enzyme deactivation by an accumulation of H_2_O_2_ above 1 mM, hybrid catalysts are tested under lower stirring speed (Figures S9 and S10).

The resulting hybrid chemo-enzymatic catalyst produces 1-(*R*)-phenylethanol from ethylbenzene with a measured *ee* of 97.7% (Figure S11), in the presence of only H_2_ and O_2_ gases. This *ee* is on par with previous literature reports for nonimmobilized, single-use PaDa-I,^6,25^ meaning that the proposed hybridization process conserves the enantioselectivity of PaDa-I. Figure 6 compares the production of PhEtOH by the enzyme in the hybrid catalyst and of the free enzyme, tested under the same reaction conditions. Free enzyme loading was adapted to match the maximum theoretical loading of the hybrid catalyst (2.925 μg_enz_/mg_solid_). While the overall activity of the hybrid catalyst is lower than that of the two-catalysts system, its activity remains constant throughout the 6 h reaction. Interestingly, no quantifiable amount of H_2_O_2_ was detected in the medium during the reaction, which indicates that the hybrid catalyst can regulate the presence of H_2_O_2_ even when PhEtOH production, and thus H_2_O_2_ consumption, is limited. Though it has not been formally proven, it is suspected that the lower activity of the hybrid catalyst is caused by diffusion limitations rather than lower intrinsic activity of the enzyme (Section S4). The TON over 6 h is 72,000 mol_product_ mol_enz_^–1^, averaging to a TOF of 12,000 h^–1^, considering zero-order kinetics. This is promising, as previous studies of fed-batch PaDa-I in 50% ACN showed TONs 18,800 mol_product_ mol_enz_^–1^ after 6 h and 19,000 mol_product_ mol_enz_^–1^ after 4 h with cyclohexane. These average to TOFs of 3,133 and 4,750 h^–1^ respectively.^15,65^ Additionally, kinetic benefits of coimmobilizing the enzyme and inorganic catalyst on the same microsphere (vs two separate heterogeneous catalysts) were observed, as reported in Figure S15. Finally, a “hot-filtration test” was performed, in which the hybrid catalyst was filtered and replaced by parent SD-AuPd/TS-1 (no enzyme) after 1 h of reaction. This test shows no sign of active enzyme leakage, as only the H_2_O_2_ concentration increases after replacing the hybrid catalyst with the inorganic catalyst (Figure 6).

**Figure 6:**
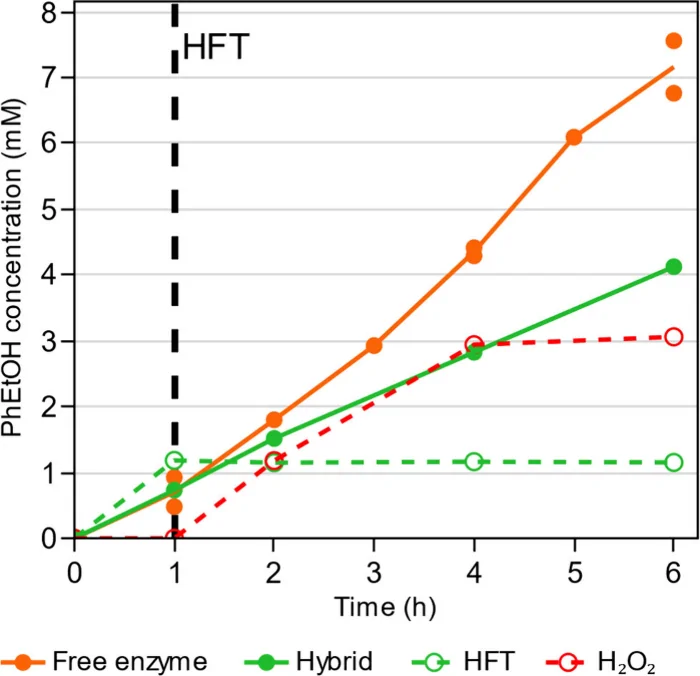
1-Phenylethanol production in cascade conditions by the free enzyme (orange) and the hybrid catalyst (green). The hot-filtration test (HFT, dashed lines) was performed by replacing the hybrid catalyst with pristine SD-AuPd/TS-1 after 1 h of reaction. The dashed red line represents H_2_O_2_ concentration during the HFT of the hybrid catalyst.

The leaching or deactivation of active enzymes was further assessed through the recyclability tests. The hybrid catalyst was used for 1 h and recovered, through either centrifugation or filtration and washed with fresh reaction medium, up to 4 times. When filtered between uses, the catalyst was reusable without consistent loss of activity toward PhEtOH (Figure 7). This confirms proper immobilization of the active enzymatic species. However, it was also shown that centrifugation between runs is detrimental to the enzymatic activity. As centrifugation has no effect on the activity of free enzymes, we suggest that this loss of activity could be attributed to the destruction of the microspheres or CLEAs, which would lead to the leaching of active enzymes between the runs. The gradual increase in H_2_O_2_ production after each cycle is attributed to the decrease in catalyst concentration due to the loss of material between runs: as H_2_O_2_ production is not catalyst-limited (*vide supra* and Figure S7); only its consumption by PaDa-I is diminished by the decrease in hybrid catalyst concentration.

**Figure 7:**
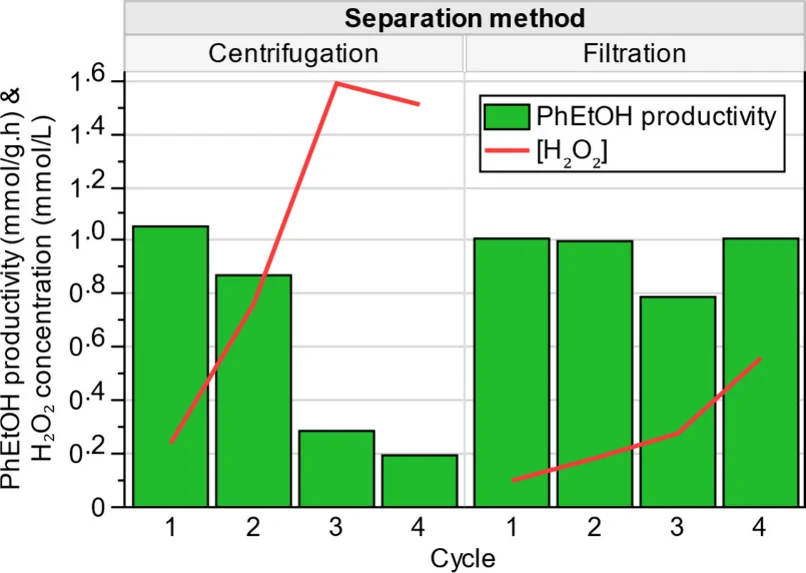
Reusability of the hybrid catalyst over 4 successive cycles. Results are compared between two recovery methods: centrifugation (left) and filtration (right).

### Spatial Mapping of the HCEHC

The spatial localization of the enzyme was confirmed by confocal microscopy. Using a hybrid catalyst produced from H6-PaDa-I in lieu of the commercial PaDa-I, it is possible to selectively label the enzyme with an anti-H6 fluorescent antibody. The images in Figure 8 show a clear difference in distribution of fluorescent probe between the control (pristine SD-AuPd/TS-1, Figure 8A,C) and the hybrid catalyst (Figure 8B,D). The control sample displays a weak signal at the surface of the material due to the nonspecific adsorption of the fluorescent antibody on the zeolite and/or gold NP. The microspheres appear in the form of hollow circles on the 2D image or hollow spheres in 3D. Conversely, the stronger signal of the hybrid materials appears as solid disks and spheres, which indicates the presence of the enzyme retaining the antibody inside the hollow particles. Moreover, the solid walls of SD-AuPD/TS-1 are also less visible on the hybrid catalyst than on the control sample, meaning the antibody was drawn away from the support and into the sphere’s cavity. This supports the hypothesis that the active PaDa-I is mostly located inside the hollow spheres and that the activity of the catalyst is not reliant on weakly adsorbed species at the surface of the inorganic catalyst.

**Figure 8:**
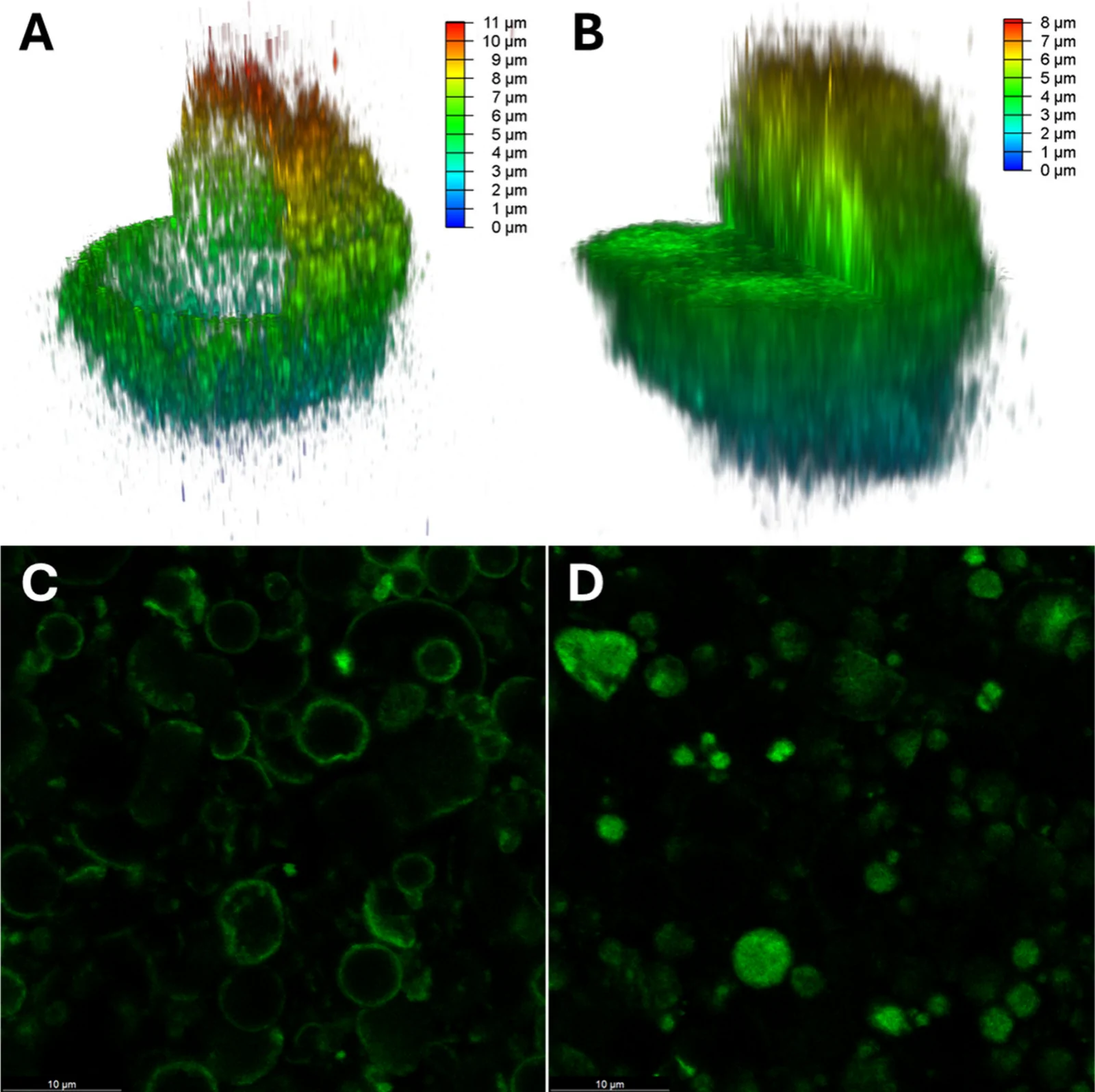
Confocal microscopy images using Alexa Fluor 488 conjugated 6x-His Tag monoclonal antibody as probe. Top: 3D resolution of an isolated microsphere of control sample (A) and hybrid catalyst (B). Bottom: overview of the particles of control sample (C) and of hybrid catalyst (D).

## Conclusion

In this work, we demonstrated the creation of a hybrid chemo-enzymatic heterogeneous catalyst by combining commercially available UPO (PaDa-I) and a Au–Pd catalyst shaped via spray drying. The modified inorganic catalyst was shown to have increased productivity over the pristine material, likely due to its lower activity toward H_2_O_2_ degradation. We also demonstrated that the use of an enzyme in the form of a crude extract was beneficial to the formation of stable CLEAs, rather than using a purified PaDa-I. The choice of the precipitating agent used for the CLEA process was shown to be critical to both the activity and the stability of the resulting material, with ethyl lactate offering the best overall results.

The resulting hybrid chemoenzymatic heterogeneous catalyst retains stereoselectivity (*ee* = 97.7%) and activity, without leaching, for at least 6 h. Under mild conditions (room temperature, 3 bar (10% H_2_, 90% air)), it can reliably produce around 1 mmol g^–1^ h^–1^ of 1-(*R*)-phenylethanol from ethylbenzene. The location of the enzyme in the material was spatially resolved through confocal microscopy, confirming its presence inside the microspheres of the inorganic catalyst. Such a spatial configuration enables the total recovery of the active hybrid material through filtration and its reuse.

The rate-limiting step of the system currently being the activity of the biocatalyst, scale-up of this system to preparative scale would mainly introduce engineering challenges. Indeed, as the biocatalytic step is performed within the catalyst microsphere which produces H_2_O_2_, the production of the latter from molecular gases would likely be the main source of change. With increasing reactor volume, H_2_ and O_2_ transfer rate would expectedly decrease^66^ (Section S2), lowering the production of H_2_O_2_. This would however have no ill effect on the enzyme and may, at worse, reduce the overall catalytic activity—if EB remains sufficiently mixed. The main challenge would be to match the bio- and chemocatalyst activities by changing operational parameters such as stirring rate, reactor shape or, if necessary, gas and liquid compositions and pressures or the addition of sparging.

This chemoenzymatic system arguably represents a promising approach for greener chemical processes in enantioselective hydroxylation reactions. Though this should be substantiated via rigorous green metric calculations, the concept makes important steps in the right direction: high atom economy (no sacrificial substrate), preservation of enzyme activity, reuse of the enzyme, and avoidance of the anthraquinone process.

## Supplementary Material



## Notes

Methods, materials and procedures, solid material characterization, activity of the inorganic catalyst, optimization of the hybrid catalyst formulation and operation parameters, insights on hybrid catalyst activity, enzyme production information, discussion on safety and green metrics (PDF)
