# Supplementary material for: Hybrid Chemo-Enzymatic Heterogeneous Catalyst for Green and Enantioselective Hydroxylation
Source: ACS Cent Sci. 2026 Aug 27;12(9):1385–93. doi: 10.1021/acscentsci.6c00674 (PMC13614060; doi:10.1021/acscentsci.6c00674)
Supplement: Supplementary file 1 [file oc6c00674_si_001.pdf]

Supporting information

Hybrid Chemo-Enzymatic Heterogeneous Catalyst for Green and Enantioselective Hydroxylation

Marty Kinnaer[1], Justin Ovaert[1,2], Valentine Simar[1], Zuzana Hlavenková[3], David Cannella[2], Damien P. Debecker*[1]

[1] : Institute of Condensed Matter and Nanosciences (IMCN), Université catholique de Louvain (UCLouvain), Place Louis Pasteur, 1, 1348 Louvain-la-Neuve, Belgium
[2] : PhotoBioCatalysis unit – Université libre de Bruxelles, 165/61 - Avenue F.D. Roosevelt, - 1050 Belgium
[3] : Central European Institute of Technology, Masaryk University, Žerotínovo nám. 617/9, 601 77 Brno, Czech Republic

* damien.debecker@uclouvain.be

Table of contents

Experimentals S3
Catalysts preparation S3
TS-1 synthesis S3
AuPd wet impregnation – AuPd/TS-1 .... S4
Hollow spheres preparation – SD-AuPd/TS-1 .... S4
Enzyme preparation S4
Cloning, production, and purification of a His_6-tagged version of PaDa-I S5
Preparation of Cross-linked enzyme aggregates (CLEAs) S6
Hybrid chemoenzymatic heterogeneous catalysts preparation by CLEAs formation in the hollow catalyst spheres S6
Catalytic testing S7
Inorganic catalysts: H_2O_2 production S7
CLEAs: enzymatic asymmetric hydroxylation of ethylbenzene S7
Chemo-enzymatic asymmetric hydroxylation of ethylbenzene in cascade conditions S8
Hot filtration tests and catalyst reusability S8
Enzymatic activity of free enzyme and hybrid catalyst S9
Deactivation of PaDa-I by H_2O_2 .... S9
Analytical S10
GC S10
H_2O_2 quantification S10
Scanning electron miscroscopy (SEM) S10
Scanning transmission electron microscopy – energy dispersive X-ray spectroscopy (STEM-EDS) S11
Confocal microscopy S11
Chiral HPLC S12
Inductively coupled plasma optical emission spectroscopy (ICP-OES) S12
X-ray photoelectron spectroscopy (XPS) S12

High resolution nitrogen physisorption S12
Supporting results S13
S1: Inorganic catalyst characterization S13
S2: Activity of the inorganic catalyst S17
S3: Optimization of the Hybrid Catalyst formulation and operation parameters S18
S4: Insights on hybrid catalyst activity S21
S5: Enzyme production S24
pPICZB-AaeUPO*-CHis6 : genetic sequence S24
SDS-PAGE S27
S6: Discussion on safety and green metrics S27
References: S28

Experimentals

Catalysts preparation

TS-1 synthesis

The TS-1 support hydrothermal synthesis was adapted from the literature[1–3]. 0.55 g of titanium isopropoxide (TiiP) was solubilized in 5 g of isopropanol (iPOH) (solution A). In a separate vessel, 22.5 g of tetraethylorthosilicate (TEOS) were mixed with 9.84 g of distilled water and 16.51 g of tetrapropylamine hydroxide (TPAOH) (40 % w/w) to form solution B. After stirring to homogeneity, solution A was added dropwise to solution B under vigorous stirring. The resulting solution was stirred for an additional 15 minutes. Then, a solution containing 5.47 g of TPAOH (40 % w/w) diluted in 38.28 g of distilled water was added. The initial gel composition was 1 SiO_2/0.018 TiO_2/0.40 TPAOH/32 H_2O. The resulting solution was then stirred for 3 h at 75 °C to evaporate the alcohol. The resulting gel was diluted with 30 g of distilled H_2O and transferred to a Teflon-lined stainless-steel autoclave. Crystallization was carried out at 160 °C for 24 h. After rapid cooling, the resulting precipitate was recovered by centrifugation and washed thoroughly with distilled H_2O until neutral pH, dried under static air at 130 °C overnight and finally calcined under static air at 550 °C for 5 h (5 °C/min).

AuPd wet impregnation – AuPd/TS-1

The impregnation of gold and palladium nanoparticles was carried out following a well reported procedure of anion-assisted wet impregnation [4–6]. In a typical synthesis, a solution of 9.92 g/L of $PdCl_2$ was prepared in 0.58 M HCl, along with a solution of 10.2 g/L $HAuCl_4.3H_2O$ in distilled water. The required amount of each solution was then added to a round-bottom flask and diluted with distilled water to reach a total volume of 8 mL per gram of final solid. The final Cl^- concentration is 0.085 M. After heating to 60 °C under stirring, the solid support was slowly added to the solution. The resulting slurry was stirred for an additional 15 minutes at 60 °C to ensure homogeneity. Then, water was evaporated overnight under stirring at 90 °C. The resulting solid was ground, sieved and stored in a desiccator at room temperature.

Hollow spheres preparation – SD-AuPd/TS-1

The hollow inorganic catalyst spheres were obtained via a spray-drying method previously developed in our group[1]. First, a 10 % w/w suspension of the desired solid was prepared using distilled H_2O. The suspension was sonicated for 10 minutes and thoroughly stirred to break any aggregates of the solid particles. In the meantime, a silica binder solution was prepared. In a typical synthesis, 4.9 g distilled H_2O, 1.19 g TPAOH (40 % w/w) and 3.26 g TEOS were mixed and stirred for 1 h, then heated to 80 °C for 6 h in a closed vessel. Then, 0.94 g of Pluronic® F-127 was added and further stirred for 1 h. The resulting composition of the binder solution was 1 SiO_2/ 0.15 TPAOH/ 0.005 F127/ 20 H_2O. Before spraying, the inorganic catalyst suspension was mixed for 30 minutes with 2.47 g of binder solution per gram of solid. The resulting mixture was then sprayed with a Büchi Mini Spray Dryer B-290 with an air pressure of 5 bar, maximim flow rate and set at 90 % aspiration and 15 % pump rate. The aerosol was dried at 80 °C by passing through a pre-heated glass reactor and collected in a cyclone separator. The resulting powder was then dried at 70 °C under vacuum overnight and calcined at 550 °C for 5 h (5 °C/min) to eliminate the organic moieties. Unless stated otherwise, all catalysts were reduced under a flow of H_2/Ar (5 %) for 3 h at 400 °C (10 °C/min) prior to use.

Enzyme preparation

Commercial PaDa-I was purchased from EvoEnzyme SL and used without modification, as a 4.5 mg/mL solution of crude extract.

Cloning, production, and purification of a His_6-tagged version of PaDa-I

The His_6-tagged version of the engineered variant r*Aae*UPO* (PaDa-I mutant) was obtained by recombinant expression in *Pichia pastoris* according to literature[7,8], with minor modifications. The synthetic gene of the C-terminal His_6-tagged engineered variant rAaeUPO* (PaDa-I mutant)[7] was obtained in the standard *Pichia pastoris* expression vector pPICZ-B (BioCat GbmH, Germany). Upon transformation of electrocompetent *P. pastoris* X-33 with the linearized plasmid and selection of the highest UPO-expressing transformants, the recombinant enzyme was expressed in a 6 L glass-vessel bioreactor (Minifors 2, Infors-HT, Switzerland) following a procedure adapted from Pichia Fermentation Process Guidelines[9] and a Short protocol from Eppendorf[10]. Briefly, the glass vessel containing 2 L of Basal Salt Medium (42,9 g/L KH_2PO_4, 5.17 g/L $(NH_4)_2SO_4$, 14.33 g/L K_2SO_4, 0.6 g/L $CaSO_4.2H_2O$, 5.71 g/L $MgSO_4.7H_2O$, 0.5 ‰ (v/v) H_2SO_4 (98 %), 2 % (w/v) glycerol) supplemented with 0.03 % of antifoam (Struktol J650) was autoclaved. After autoclaving, 8 mL of PTM4 trace elements (2 g/L $Cu_2SO_4.5H_2O$, 0.08 g/L NaI, 3 g/L $MnSO_4.4H_2O$, 0.2 g/L $Na_2MoO_4.2H_2O$, 0.02 g/L Boric acid, 0.5 g/L $CaSO_4.2H_2O$, 0.5 g/L $CoCl_2$, 7 g/L $ZnCl_2$, 22 g/L $FeSO_4.7H_2O$, 0.2 g/L Biotin) were added aseptically. Upon equilibration of the medium and adjustment of the pH with a solution of 28 % ammonium hydroxide to reach the initial bioprocess operating conditions (30 °C, pH 5), the fermentation process was initiated by inoculating the medium with 200 mL of *P. pastoris* preculture (OD600 > 2) grown at 30 °C and 230 rpm in Buffered Glycerol Complex Medium (BMGY) for 18 h in two separate 500 mL baffled flasks (containing each 100 mL). The batch was run at 30 °C, until glycerol was completely consumed, marked by a sharp increase in the Dissolved Oxygen concentrations (DO). This sharp increase in DO initiated the glycerol feed, containing 99.5 % glycerol supplemented with 0.8 % (v/v) PTM4 trace elements. The glycerol fed-batch phase was continued for 21 hours, with a feed flowrate of 6.5 – 16.2 mL/h. After the glycerol fed-batch phase, a casamino acids solution was injected aseptically to reach a final concentration of 3 g/L, and the temperature was set to 23 °C. Then, the methanol feed containing 99.5 % methanol supplemented with 0.8 % PTM4 trace elements was initiated and maintained at a flowrate of 6.5 mL/h for 96 h. Throughout the whole process, the DO was maintained above 20 % by automatically adjusting stirring speed and air sparging flowrate, pH was maintained at a value of 5 using 28 % ammonium hydroxide and samples were taken regularly to monitor the glycerol/methanol concentration, wet biomass and UPO activity.

Cells were separated from the medium by centrifugation, and the supernatant was filtered using a Nalgene™ Rapid-Flow™ 75 mm Filter unit (Thermo Scientific) and supplemented with Tris-

HCl 1 M (pH 7.5) to a final concentration of 50 mM. The recombinant protein was then purified using a Ni^{2+}-affinity chromatography column (HisPrep^{TM} FF 16/10, Cytiva) mounted on an ÄKTA Start^{TM} chromatography system (Cytiva) as follows: prior to sample application, the column was equilibrated with 40 mL of Buffer B (50 mM Tris-HCl (pH 7.5), 250 mM NaCl, 500 mM Imidazole), followed by 100 mL of Buffer A (50 mM Tris-Hcl (pH 7.5), 250 mM NaCl); upon sample injection and binding, the column was washed with 180 mL of a 96:4 (% v/v) mixture of Buffers A and B respectively; the recombinant protein was then eluted by applying a gradient of Buffer B from 4 % up to 100 % over 180 mL. Collected elution fractions containing the recombinant protein (detected using the UV detector) were concentrated and the buffer was exchanged to Tris-HCl 50 mM (pH 7.5) by using Vivaspin 20 centrifugal concentrators (10 kDa MWCO, Sartorius).

Complete protein and gene sequences gene map and SDS-PAGE are available in Section S5.

Preparation of Cross-linked enzyme aggregates (CLEAs)

Non-entrapped CLEAs were prepared by precipitating 100 µL of a 2.25 mg/mL enzyme solution (PaDa-I or H6-PaDa-I) with 1 mL of precipitating agent (ethyl lactate (EL), acetone, isopropanol (iPOH), acetonitrile (ACN)) or DI water (as a negative control). After homogenization, 18.7 µL of glutaraldehyde (GAH) (25 %) were added to reach a GAH concentration of 50 mM. The resulting solution was homogenized and left to react at room temperature for 2 hours. The resulting CLEAs were centrifuged briefly at 2,000g and washed three times with 1 mL DI water.

Hybrid chemoenzymatic heterogeneous catalysts preparation by CLEAs formation in the hollow catalyst spheres

The HCEHCs were synthesized via combination of SD-AuPd/TS-1 inorganic catalyst and UPO following the method previously developed in our group [1] adapted with specific information on UPO immobilization from the literature[11] and further optimized. The enzyme is immobilized on the support via entrapment and cross-linking inside the hollow microspheres via a two-step procedure: 1) 500 mg of reduced SD-AuPd/TS-1 is impregnated with 0.9 mL of a 2.25 mg/mL solution of UPO (PaDa-I or H6-PaDa-I). The suspension was gently stirred using a rotary agitator for 15 minutes at 50 rpm. Vacuum was then applied to remove air from the solid material, effectively forcing the enzyme solution inside the microsphere cavities. The excess liquid was then removed by centrifugation at 2,000g, to limit the formation of CLEAs outside the microspheres in the next step. 2) 9 mL of precipitating agent (ethyl lactate or isopropanol)

was added to the pellet, and the suspension was stirred gently in the rotary agitator. After 30 minutes, 170 µL of GAH was added to the suspension (25 %, final concentration of 50 mM) which was further stirred for another 2.5 h. Then, the solution was left to rest overnight at 4 °C. Finally, the powder was recovered by centrifugation and washed with distilled water until no enzyme was present in the supernatant (Bradford assay). This typically corresponds to 4 washes with 10 mL of water. The resulting catalyst was stored at 4 °C in Tris-HCl buffer (pH 7.6) and is ready to use without further activation step.

Catalytic testing

Inorganic catalysts: H_2O_2 production

The activity of the inorganic catalyst was assessed via their 1-hour production of H_2O_2. Reactions were carried out in a 25 mL Büchi tinyclave® glass reactor loaded with 25 mg of catalyst and 25 mL of freshly prepared reaction medium. The charged autoclave was then purged 3 times with dry air then filled with 2.7 bar of dry air and 0.3 bar H_2. Pressures are given as absolute pressures, the initial pressures of H_2 and O_2 are 0.3 bar and 0.57 bar respectively, outside of explosive conditions.[4–6,12–14] Reactions are performed in a closed system at 22 °C under vigorous stirring, no gas is supplied during the reaction. After 1h, the reaction medium is sampled and filtered through a 0.22 µm mesh, and H_2O_2 production is measured via a colorimetric assay with 15 % w/v titanium (IV) oxysulfate.

CLEAs: enzymatic asymmetric hydroxylation of ethylbenzene

Immediately after preparation, CLEAs pellets were each added to 15 mL of reaction medium containing 40 % (v/v) acetone (VWR, HPLC grade 99.8 %), 60 % (v/v) Tris buffer (pH 7.6, 50 mM, Sigma-Aldrich), 20 mM Ethylbenzene (99 %, Sigma-Aldrich) and 4.5 mM 2'-hydroxyacetophenone (IST) (>98 %, TCI, internal standard, weighed accurately in a precise volume). Positive controls were performed by replacing the CLEAs with equivalent amount of free enzyme (225 µg). Reaction was started by adding 15.4 µL of H_2O_2 0.97 M to reach a final concentration of 1 mM. Reaction was carried out for 20 min under mild rotating agitation. Then, a 950 µL aliquot was added to 50 µL $TiOSO_4$ for colorimetric analysis. A second aliquot was quickly added to ethyl acetate (EA) for GC analysis. Both $TiOSO_4$ and EA immediately stop the reaction.

Chemo-enzymatic asymmetric hydroxylation of ethylbenzene in cascade conditions

The typical reaction medium was prepared by adding 60 µL of ethylbenzene (99 %, Sigma-Aldrich) in 12 mL acetone (VWR, HPLC grade 99.8 %). Once homogenized, the mixture is diluted with 18 mL Tris HCl (50 mM, pH 7.6) to reach a ratio of acetone/water of 2/3 (v/v). Reactions were carried out in a 25 mL Büchi tinyclave® glass reactor loaded with 25 mg of solid catalyst and 25 mL of freshly prepared reaction medium. 20 µL of 2'-hydroxyacetophenone (>98 %, TCI) were accurately weighed in the reactor as internal standard. When testing free UPO, 73 µg of UPO are also added to the reactor (PaDa-I: 16.25 µL, 4.5 mg/mL. H-6-PaDa-I: 22 µL, 3.3 mg/mL). Reactions are performed in a closed system at 22 °C under vigorous stirring. After 1 h, 2 h, 4 h of reaction, the reactor is opened, and 200 µL are sampled for GC analysis. When performing 6 h kinetic tests, each sampling implies opening the reactor for sampling and repressurizing it. We acknowledge that the measurement can thus impact the kinetics of the reaction; only kinetics measured at the same reaction times are compared in this study. After each 200 µL sampling, the reactor is repressurized to initial conditions to resume reaction (purged 3 times with dry air then pressurized with 2.7 bar of dry air and 0.3 bar H_2). Pressures are given as absolute pressures, the initial pressures of H_2 and O_2 are 0.3 bar and 0.57 bar respectively, outside of explosive conditions.[4–6,12–14] The Samples are extracted with 200 µL of ethyl acetate. The organic fraction (top) is used for GC analysis. The aqueous fraction (bottom) is used for H_2O_2 quantification. After 6 h, 500 µL is sampled for GC analysis, and 1.5 mL for precise H_2O_2 quantification. Blank tests were performed by removing either the inorganic catalyst, the enzyme, or H_2 from the system.

Hot filtration tests and catalyst reusability

The robustness of hybrid catalysts is tested by a hot filtration test to monitor active enzyme leaching, and reusability tests to assess the retention of catalytic activity through repeated reaction and recovery cycles.

Hot filtration tests of the hybrid catalysts were performed in the same initial conditions as regular cascade reaction tests. After one hour, the reaction medium is filtered through a 0.22 µm mesh to remove the catalyst, and samples of the filtrate are taken for colorimetric and GC analysis. Then, 1 mg/mL of pristine inorganic catalyst (without enzyme) is added to the reactor, to resume the production of H_2O_2 in the medium. This enables the detection of active enzymes leached in the reactor, regardless of the consumption of H_2O_2 during the first hour of reaction.

The reaction is carried out for 6 hours, similarly to a standard catalytic test, with samplings after 2 h, 4 h, and 6 h.

Catalyst reusability was performed in the same conditions as regular catalytic tests. After one hour, the catalyst is recovered and washed 3 times with a water-acetone mixture (3/2 v/v) before being introduced in a fresh reaction medium for a new reaction cycle. This process is repeated up to four consecutive reaction cycles of 1 h. Two methods of catalyst recovery were tested: filtration (A) and centrifugation (B). (A): the reaction medium is filtered through a 0.22 µm mesh to recover the catalyst, and samples of the filtrate are taken for colorimetric and GC analysis. The filtered catalyst is then washed by passing 3x5 mL of water-acetone mixture (3/2 v/v) through the filter. Then, the catalyst is recovered and added to the reactor loaded with 25 mL of fresh reaction medium. (B): the reaction medium is centrifuged for 5 minutes at 10.000g to form a coherent pellet. To wash the pellet, it is then resuspended in 15 mL of water-acetone mixture (3/2 v/v) and centrifuged. This process is performed three times. Finally, the pellet is resuspended with 25 mL of fresh reaction medium. In both processes A and B, samples are taken at the start and end of each reaction cycle to quantify H_2O_2 and organic species.

Enzymatic activity of free enzyme and hybrid catalyst

Enzymatic activities in the hybrid catalyst and free enzyme were assessed through the oxidation of EB to PhEtOH in the presence of 1 mM of exogenous H_2O_2 with varying concentrations of (bio)catalyst. Reactions were performed in lidded 24-well plates using a water/acetone mixture (2/3 v/v) as solvent buffered by Tris-HCl at pH 7.6. Reaction were started by the addition of required amount of (bio)catalyst (0-1 mg_{solid}/mL or 0.675-3.375 μg_{enz}/mL) to a solution containing 1 mM H_2O_2, about 4.5 mM IST (weighed precisely) and 20 mM EB. (Bio)Catalyst concentrations were chosen to match enzyme concentration ranges. Samples were taken at reaction start and after 10 minutes for GC analysis and H_2O_2 measurements. An additional reaction was carried out with 0.4 mg_{solid}/mL for 30 minutes with samplings at 0, 5, 10, 20, 30 minutes to assess the kinetics of the reaction by the hybrid catalyst.

Deactivation of PaDa-I by H_2O_2

The deactivation of PaDa-I by H_2O_2 exposure was assessed in 48 well plates *via* the oxidation of 5-nitro-1,3-benzodioxole (TCI, >98.0 %) (NBD) in the presence of 1 mM H_2O_2 after incubation at various concentrations of H_2O_2. All solutions were prepared in a water/acetone mixture (2/3 v/v) buffered at pH7.6 by Tris-HCl. 7.3 ng of PaDa-I were incubated 15 minutes in solutions containing 0-2.5 mM of H_2O_2 (400 µL total volume), creating solutions A_i (A_1, A_2,

…). In the meantime, substrate solutions (solutions B_i) were prepared: to 1000 µL of saturated NBD solution, dilute H_2O_2 (10 mM) was added to reach a final concentration of 0-1.67 mM after volume adjustment to 1.4 mL. After incubation, 600 µL of solution B_i was added to solutions A_i to start the reaction. Solutions A_i&B_i were matched to reach a reaction volume of 1000 µL and an initial concentration of 1 mM H_2O_2. PaDa-I concentration was 2.925 µg/mL. Absorbance was read continuously at 425 nm for 10 minutes to monitor reaction kinetics and after 5 hours. Negative controls were performed simultaneously in wells without PaDa-I to measure non-enzymatic NBD degradation. Absorbances were corrected using the corresponding negative control.

Analytical

GC

GC analyses were carried out on a Bruker SCION 456-GC. The system was equipped with a Scion-1 column (30 m x 0.25 mm x 0.25 µm), the injector is set at 250 °C with a split ratio of 10. 1 µL of sample is injected with an automatic sampler. The detector used was an FID detector at a temperature of 250 °C using 300 mL/min of air, 30 mL/min of hydrogen, and 20 mL/min of helium as make-up gas. The head of the column was kept at a constant pressure of 10 psi.

H_2O_2 quantification

H_2O_2 is quantified via a colorimetric method. The samples are filtered through a 0.22 µm mesh to remove any solid residue. 3 different methods have been developed based on the quantity of available sample. In each method, 50 µL of 15 % (w/w) titanium (IV) oxysulfate is first added into semi-micro VWR polystyrene cuvettes. Method 1: 950 µL of filtered sample are directly added to the cuvette. Method 2: 100 µL of filtered sample are added to the cuvette, and volume is adjusted to 1000 µL with DI water. Method 3: 100 µL of the aqueous phase from liquid-liquid extraction with EA is added to the cuvette, and volume is adjusted to 1000 µL with DI water. Absorbance is measured at 405 nm using a Thermo Fisher scientific Genesys 10S VIS spectrophotometer. Three distinct calibration curves have been made, one for each sampling method.

Scanning electron miscroscopy (SEM)

High resolution SEM analyses were performed by CEITEC MU. The images were acquired using a Thermo Fisher Scientific Helios 5 Hydra system equipped with a Through-Lens Detector (TLD). Imaging conditions were set to an accelerating voltage of 1.4 kV, beam current

of 800 pA, and a dwell time of 50 ns. Backscattered electrons were detected, and the final image was generated by integrating 10 individual frames.

SEM images used to determine microsphere size distribution were acquired using a JEOL 7600F at an accelerating voltage of 15 kV.

Scanning transmission electron microscopy – energy dispersive X-ray spectroscopy (STEM-EDS)

STEM-EDS measurements of SD-AuPd/TS-1 were performed by CEITEC MU using a Thermo Fisher Scientific Talos F200C instrument operating at 200 kV. The instrument was equipped with a Bruker XFlash 6T|30 EDS detector, a high-angle annular dark-field (HAADF) detector and a Thermo Fisher Scientific Ceta 16-megapixel CMOS camera. The powdered sample was dispersed in cyclohexane and 4 µL was deposited on a gold grid covered by QuantiFoil® holey carbon R2/2. This was then filtered and dried in air. STEM-EDS measurements of AuPd/TS-1 were performed at 200 kV on a Talos F200i Transmission Electron Microsocope, using VELOX software. Nanoparticle size distribution was evaluated using nanoDetect v2.2 software.[15]

Confocal microscopy

Samples were stained using Alexa Fluor® 488 His-tag specific antibody (Thermo fisher cat# MA1-21315-A488). Two solutions were prepared: solution T (BSA 2 mg/mL in PBS 0.025 M) and solution A (MA1-21315-A488 in solution T, dilution 1:50). 10 mg of sample were suspended in 24 µL of solution T and stirred 5 min at room temperature to saturate potential unspecific binding sites with BSA. Samples were then centrifuged and the supernatant replaced with 24 µL of solution A. The suspension was left stirring overnight at 4 °C. After binding, the samples were washed 5 times with TPBS (PBS 0.025 M + Tween 0.1 %) for 45 minutes and once for 2 hours. Supernatants were monitored by spectrophotometry to confirm the absence of protein and antibody. A final wash was performed with DI water to remove the surfactant.

The stained samples were resuspended in DI water and deposited in a borosilicate CELLview slide (Greiner), then left time to settle. Sample settling was required to limit movement from convective currents induced by laser irradiation. Once settled, the samples were analyzed using a Stellaris LEICA DMI8 running Las-X Version 4.7. Laser WLL was set at 85 %, emission wavelength was 580 nm and laser line was 492 nm with an intensity of 4.99 %.

Chiral HPLC

Samples for chiral HPLC were prepared by liquid-liquid extraction in an equal volume of dichloromethane. Analyses were carried out using a Waters Alliance 2690 separation module coupled to a Waters 2998 photodiode array (PDA) detector. Samples were injected (10 µL) and separated on a Daicel CHIRALPAK® IB chiral stationary phase (250 × 4.6 mm, 5 µm particle size). The mobile phase consisted of isohexane/ethanol (95:5, v/v) and was delivered under isocratic conditions at a flow rate of 1.0 $mL \cdot min^{-1}$. All separations were performed at ambient temperature, with a total run time of 10 min. Detection was achieved using the PDA detector with UV extraction at 206 nm. The retention times of (R)-1-phenylethanol and (S)-1-phenylethanol were determined using commercially available standards (TCI Chemicals) and were found to be 6.2 min and 6.8 min, respectively.

Inductively coupled plasma optical emission spectroscopy (ICP-OES)

Bulk elemental concentrations were determined using an ICP-OES (Perkin Elmer, Avio 220 and Perkin Elmer S23 Auto-Sampler) equipped with a Scott Type spray chamber and a Concentric glass nebulizer. The plasma was operated at a radiofrequency power of 1500 W with an argon flow rate of 10 L/min. Target elements were quantified at specific emission lines (Gold: 267, 595 nm, Palladium: 340, 458 nm, Silicon: 251, 611 nm and Titanium: 334, 940 nm) using an external calibration curve ($R^2 > 0.999$). Samples were prepared by dissolving roughly 10 mg (weighed precisely) overnight in a mixture of *aqua regia* (3 mL) and HBF_4 (1 mL). After complete dissolution, samples are diluted with HNO_3 (2 %) to suitable concentrations for analysis.

X-ray photoelectron spectroscopy (XPS)

Surface elemental analysis was performed using an SSX 100/206 Photoelectrons Spectrometer (Surface Science Instruments, USA) equipped with a monochromatized Al-Kα X-ray source operated at 20 mA, 10 kV. Binding energy was calibrated using the Si 2p peak at 103.5 eV. Peaks are normalized, deconvoluted and integrated on a Shirley-type baseline.

High resolution nitrogen physisorption

High resolution nitrogen physisorption isotherms were measured at 77K in a liquid nitrogen bath using a Micromeritics 3Flex. About 50 mg of powder were precisely weighed for analysis and degassed under vacuum at 150 °C overnight using a Micromeritics VacPrep 061. The samples were weighed again after degassing.

Supporting results

S1: Inorganic catalyst characterization

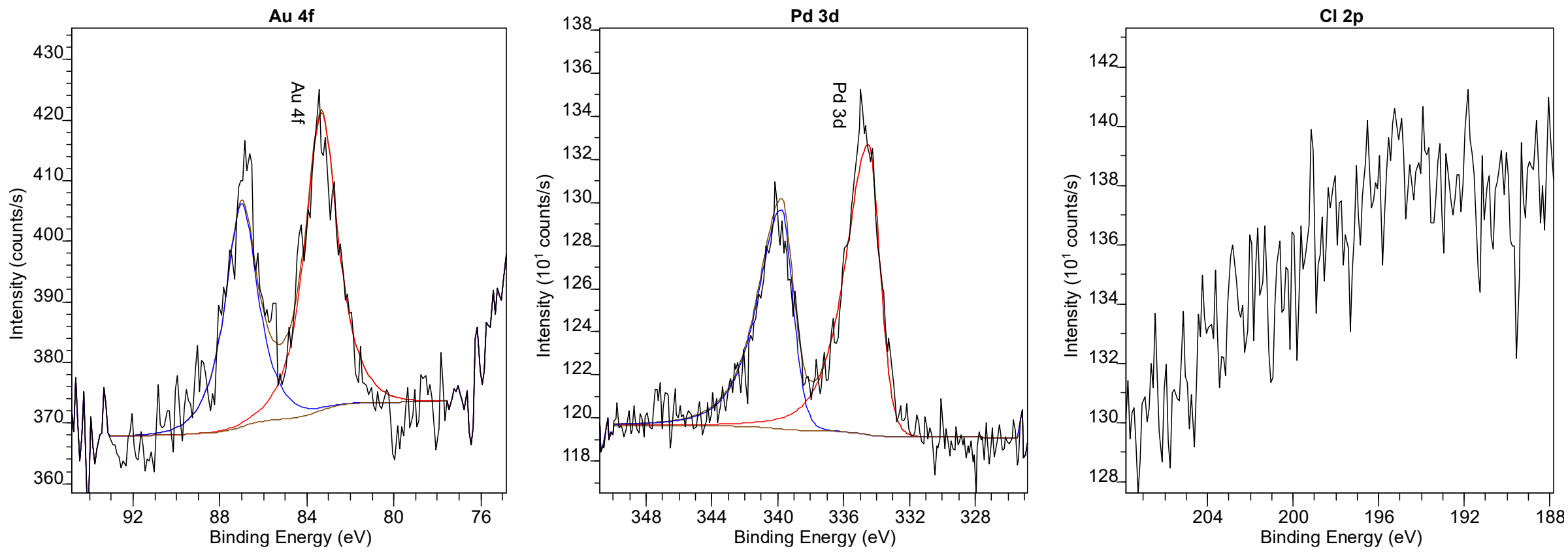

Fig. S1. XPS data of SD-AuPd/TS-1 after reduction at under 5 % H_2 at 400 °C

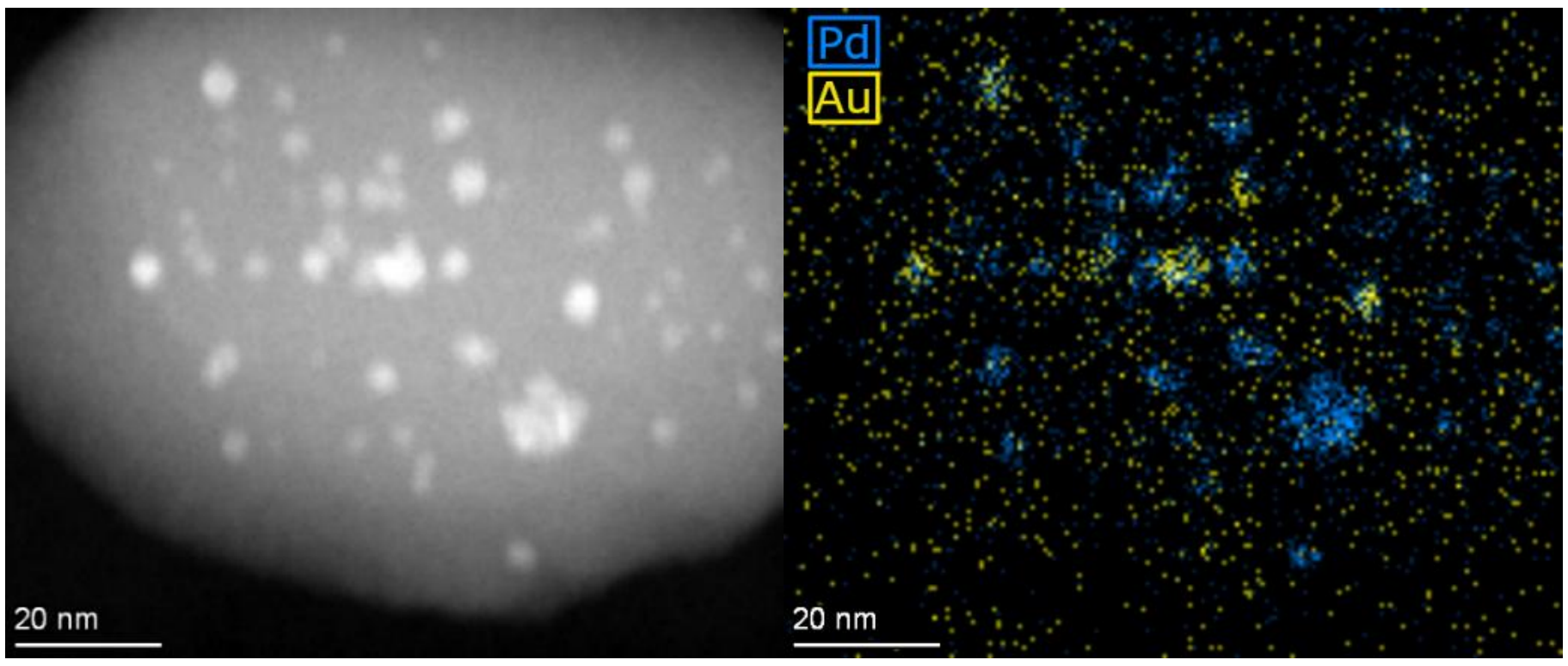

Fig. S2: STEM images of AuPd/TS-1. Left: HAADF-STEM, right: Pd and Au EDS signals

Only 6 of the visible metal particles appear to contain gold, the rest appearing to be pure Pd. These metal NP are also 5 times smaller than those of SD-AuPd/TS-1, with an average size of 3.6 nm (SD: 1.2 nm, N=194).

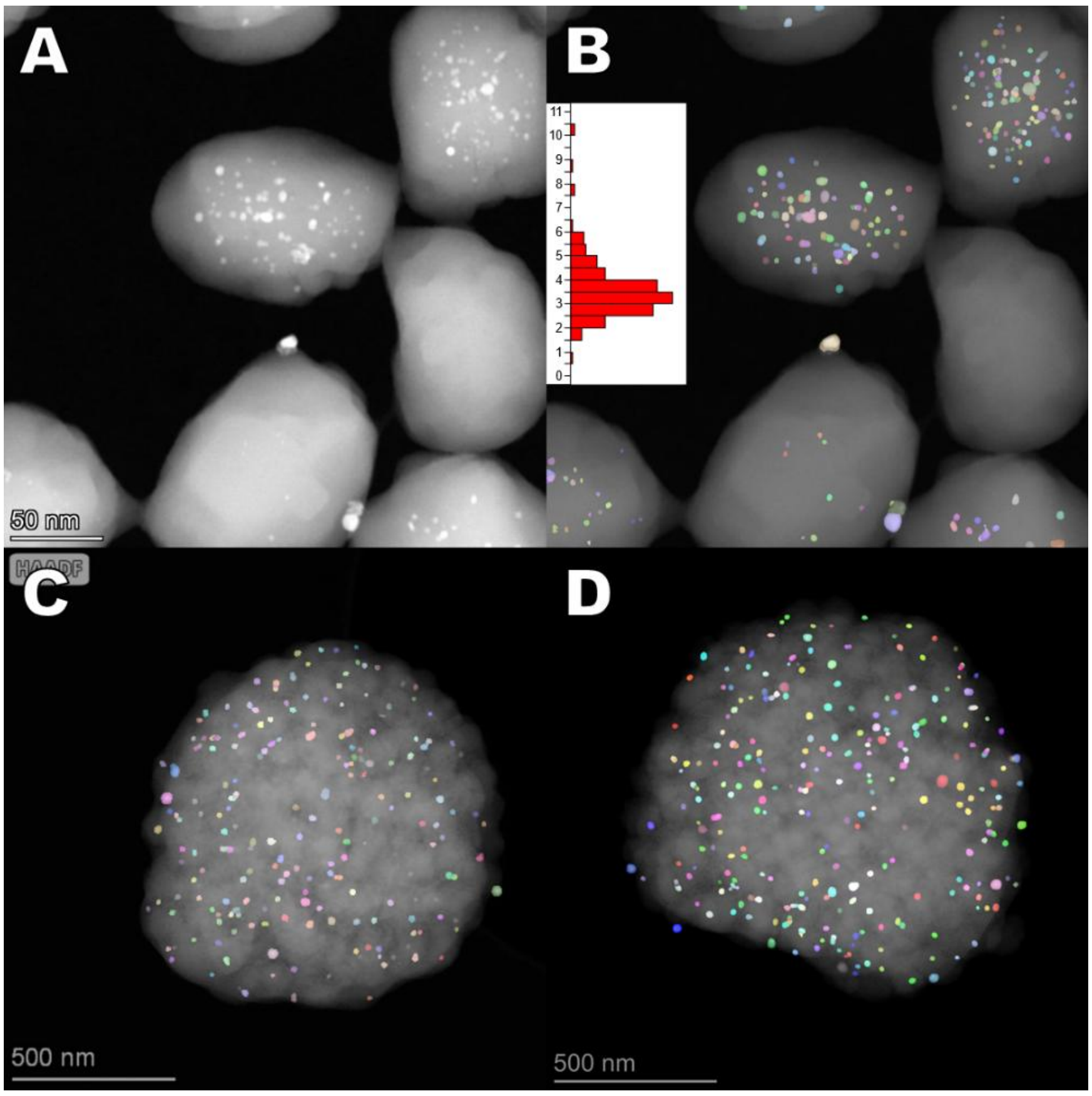

Fig. S3. STEM images used for NP counting, size was derived from the size of the colored dots. A,B: AuPd/TS-1, with size distribution. C,D: SD-AuPd/TS-1

The addition of metal NP has no significant effect on the textural properties of TS-1. As expected, the shaping induces some mesoporosity, at the cost of microporosity (Fig. S4, Table S2).

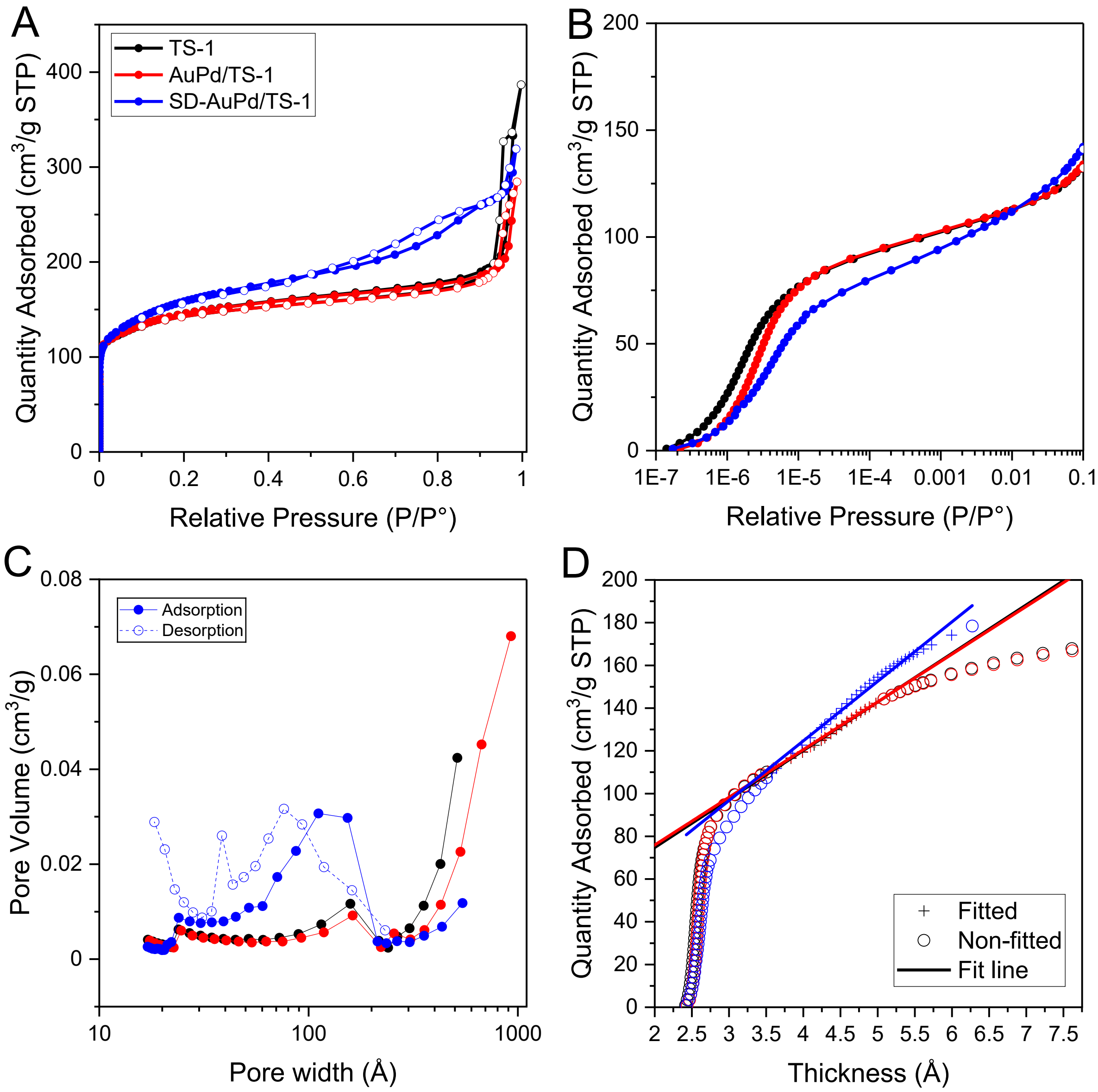

Fig. S4. N_2 physisorption data for the catalysts. A,B: Adsorption/desorption isotherm. Solid points show adsorption data, hollow points show desorption data. C: Pore size distribution using the BJH model on adsorption data. Desorption is also shown on SD-AuPd/TS-1 to account for hysteresis. D: t-plot. Thickness calculated using the Halsey model for (AuPd/)TS-1 and Harkins-Jura for SD-AuPd/TS-1.

Table S1: Textural properties measured by N_2 physisorption

Catalyst	*SSA (m²/g)(BET)*	*μ_{pore} volume (cm³/g)*	*μ_{pore} surface (m²/g)*	*Pore volume (BJH$_{ads}$) (cm³/g)*
TS-1	524	0.045	172	0.09
AuPd/TS-1	525	0.048	179	0.08
SD-AuPd/TS-1	559	0.020	127	0.20

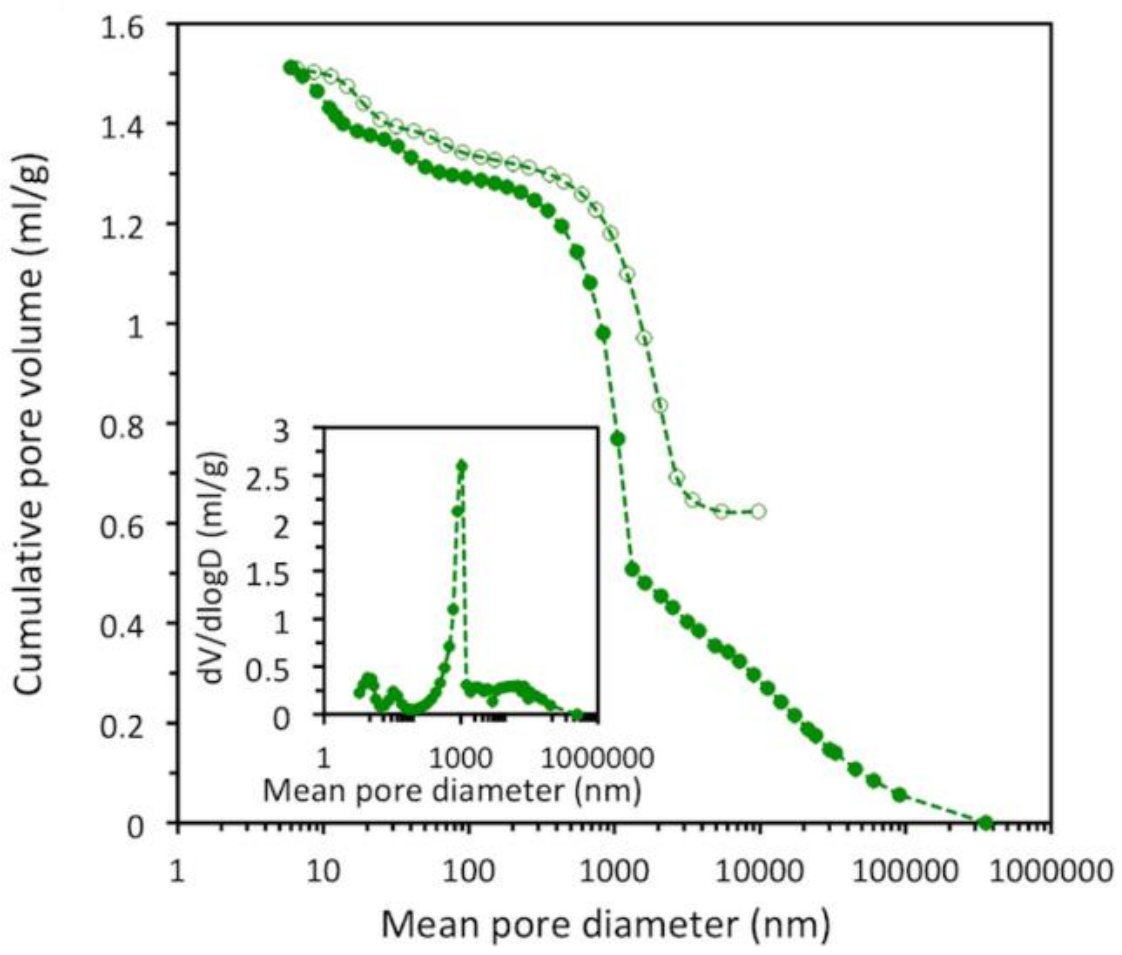

Fig. S5. Hg intrusion-extrusion isotherms for SD-TS-1. Inset shows pore size distribution. Reproduced from Smeets et al.[1]

The size distribution of the microspheres was evaluated using NanoDetect v2.2 software.[15] In accordance with our previous report on TS-1 microspheres[1], the particles follow a lognormal distribution ranging from 1 to 11 μm, with a few outliers of larger size.

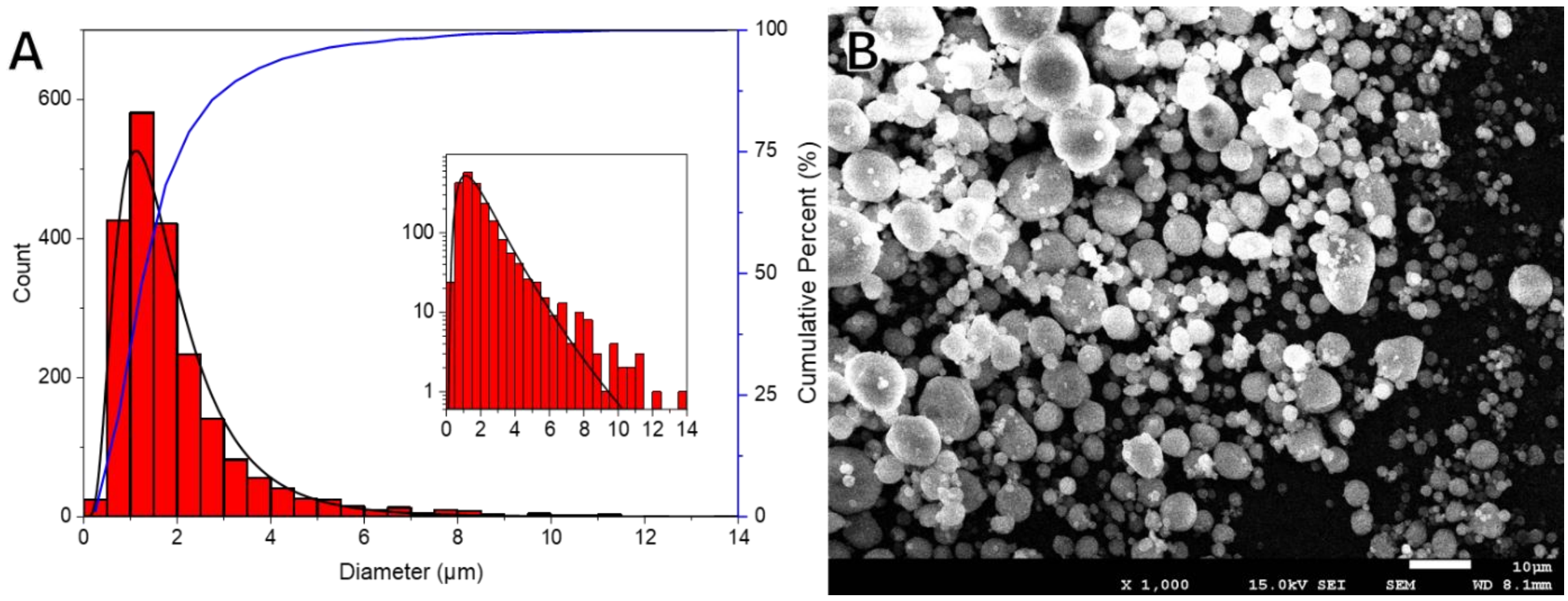

Fig. S6. A: Microsphere size distribution (N=2133). Black line shows fitted lognormal distribution, blue line shows the cumulative distribution of particles. Inset shows log scale distribution. B: Representative SEM image used for automated particle counting

S2: Activity of the inorganic catalyst

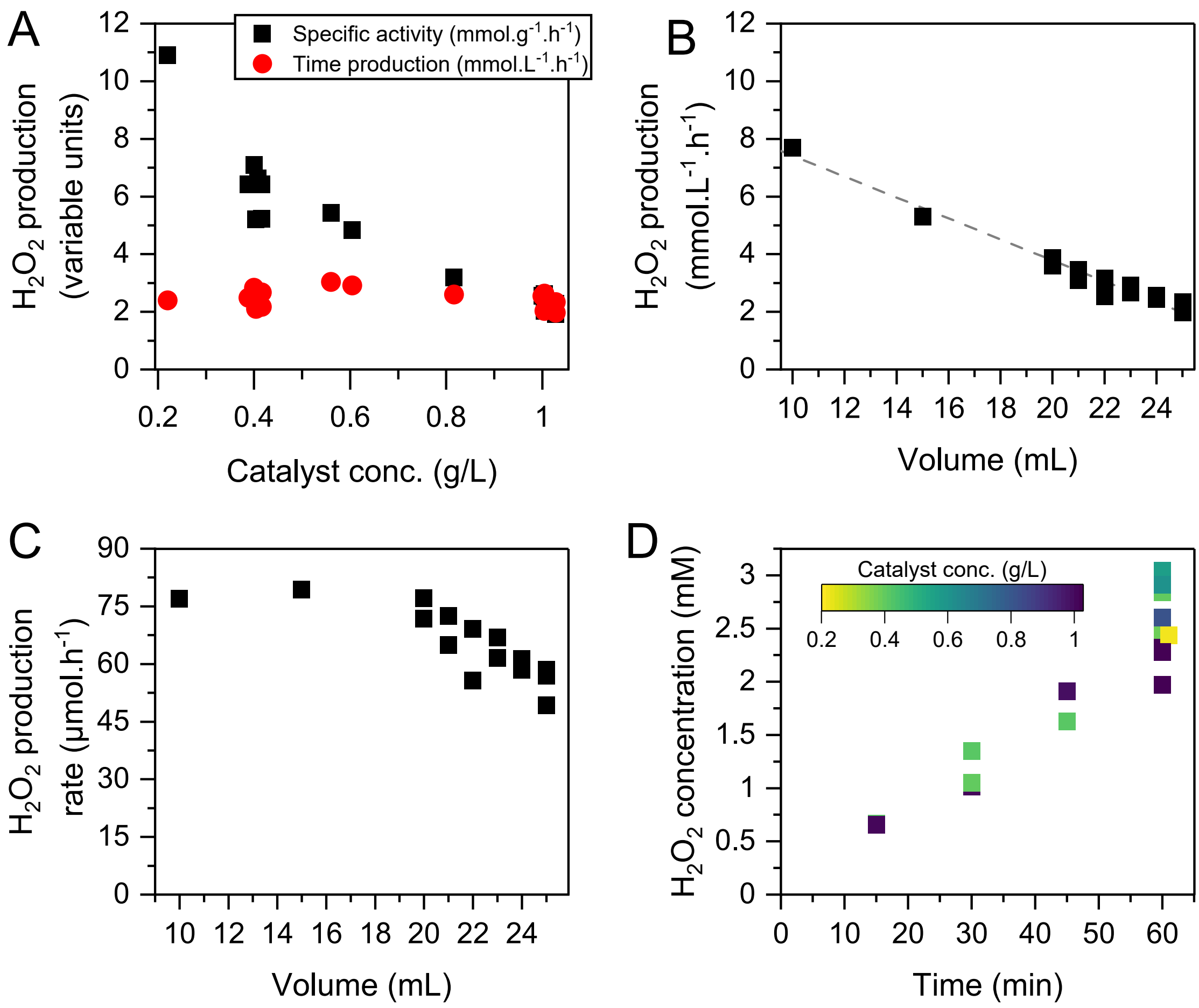

Fig. S7. H_2O_2 production by the inorganic catalyst depending on catalyst concentration (A), liquid volume (B, C), and time & catalyst concentration (D). All tests are performed at room temperature under 3 bar (10 % H_2 in dry air), data points are independent reactions.

The apparent activity of the inorganic catalyst is shown to be inversely proportional to its concentration and to the liquid volume of reaction (using the same reactor). As catalyst concentration has no impact on the concentration of H_2O_2 (Fig. S7A, red scatter), which increases linearly over time (Fig. S7D) it is evident that the limiting factor is diffusional and not catalytic. Furthermore, we show that when lowering reaction volume, the final concentration of H_2O_2 increases (Fig. S7B) but the total amount of H_2O_2 produced plateaus when reaching ~20 mL (Fig. S7C). Combined, these results indicate that the limiting factor is the diffusion of gases through the liquid phase, and that the surface-to-volume ratio is the activity-defining property of the system. This ratio can be increased by either lowering the reaction volume or increasing the stirring rate in the cylindrical reactor.

S3: Optimization of the Hybrid Catalyst formulation and operation parameters

Visual evaluation of the freshly formed CLEAs can be seen in Fig. S8 below. Aggregates formed with acetonitrile were immediately ruled out for hybrid catalyst formation as the enzyme did not precipitate into proper aggregates, but rather at the surface of the plastic, which is incompatible with a CLEA in which we aim to cross-link aggregated enzyme clusters. Recovery of those cross-linked enzymes has proven difficult as it required careful scraping of the microtube surface. Isopropanol, acetone and ethyl lactate yielded proper aggregates with both enzymes. Notably, acetone formed thick aggregates with the native PaDa-I, immediately clearing the solution before centrifugation. We believe this behavior may be deleterious to the proper formation of the hybrid catalyst as the rapid formation of large cluster may cause pore clogging in the hybrid material.

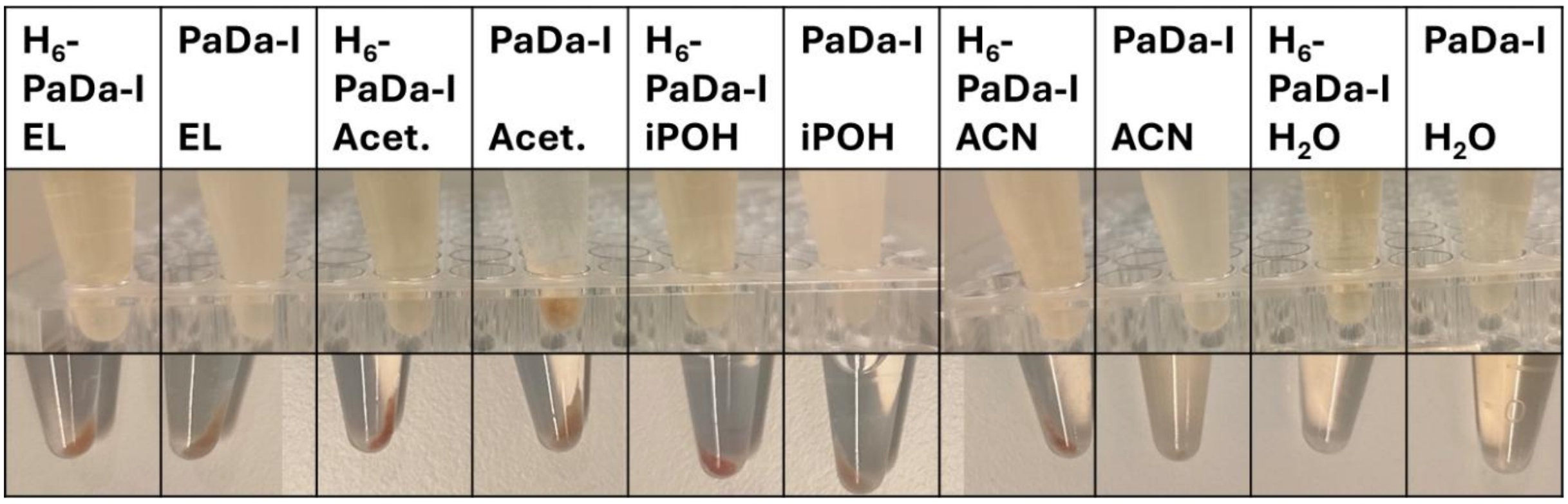

Fig. S8. Photographs of the CLEAs before (top) and after centrifugation (bottom). EL: ethyl lactate, ACN: acetonitrile, H_2O: no precipitating agent

Several HCEHC syntheses were repeated, using either Ethyl lactate (EL-HCEHC) or iPOH (iPOH-HEHC) as a precipitating agent, and PaDa-I or H6-PaDa-I. H-6PaDa-I HCEHCs were quickly ruled out as they were not reproducible and were sometimes inactive after a single day of storage at 4 °C.

iPOH-HCEHCs were shown to have higher initial activity towards PhEtOH than their EL counterparts (, left). However, the variation of activity between batches was higher than that of EL-HCEHCs. The impact of the stirring rate on HCEHCs activity was evaluated, as we showed that this parameter had an important effect on the activity of the AuPd catalyst. While higher activity is typically preferred, this system requires careful balance between the activities of the inorganic and biological catalysts to avoid accumulation of H_2O_2 and the subsequent deactivation of the enzyme. Doubling the stirring rate from 800 rpm to 1600 rpm can drastically

increase the initial activity towards PhEtOH (Fig. S9). However, this rate is not sustainable for the catalyst, which quickly deactivates as H_2O_2 accumulates in the reaction medium (Fig. S9, right).

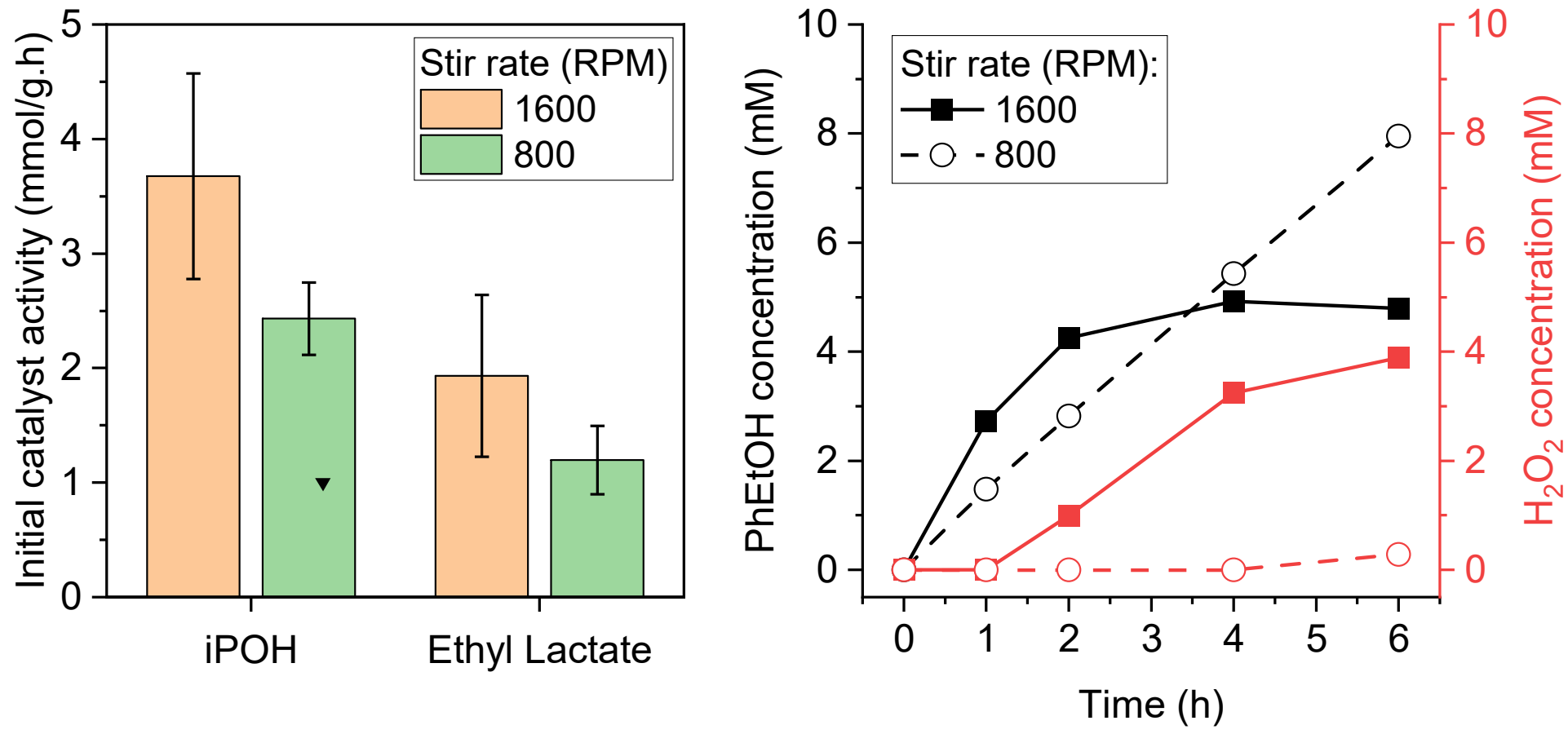

Fig. S9. left: Comparisons of the initial activities, at varying stirring rate, of HCEHCs prepared with EL or iPOH. Black triangle represents the HCEHC stabilized with $NaBH_4$. Error bars are standard deviations, not including the black triangle. Right: Reaction kinetics of EL-HCEHC at 1600 and 800 RPM.

While fresh iPOH-HEHCs display high activity, it dropped significantly after each reuse of the catalyst (Fig. S10), down to roughly 5-20 % of initial activity on the third cycle. EL-HEHCs, however, were shown to retain activity better across recyclings. Stabilizing the CLEAs in iPOH-HCEHCs by reducing the Schiff bases with $NaBH_4$[16] the resulting catalyst was far slower than counterparts (Fig. S9, left), and showed little increase in stability (Fig. S10).

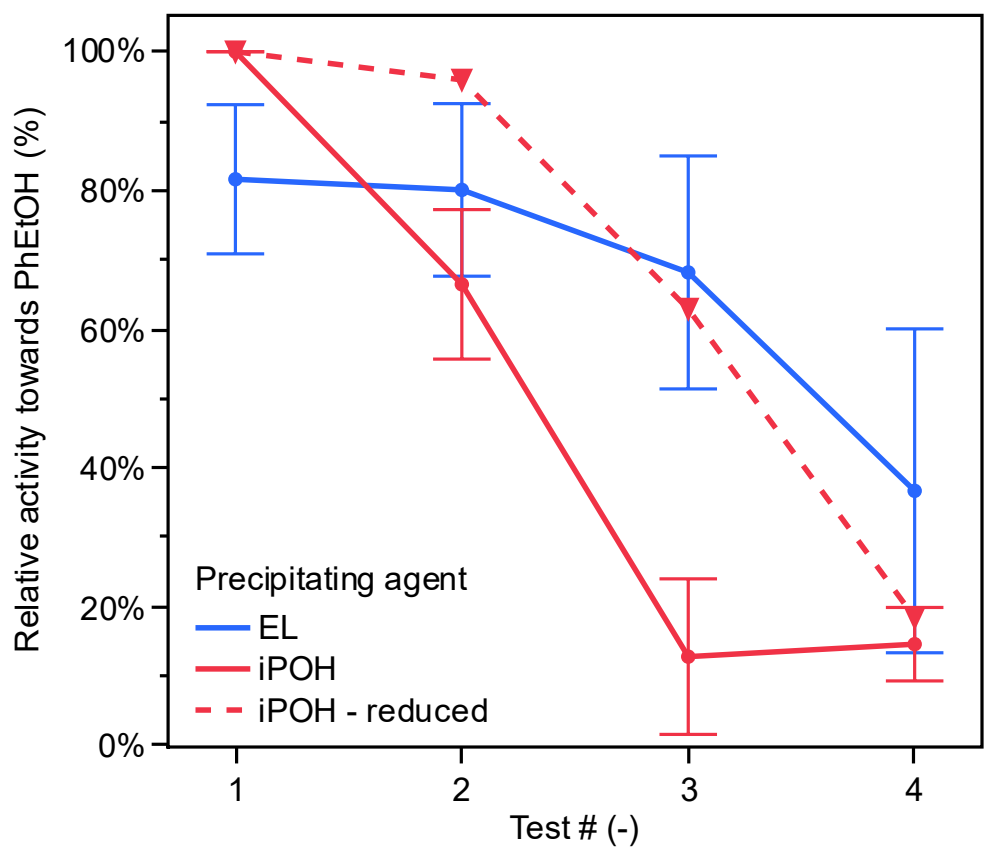

Fig. S10. Recyclability of HCEHCs prepared using EL or iPOH as a precipitating agent. Data is shown as relative activity with regards to the most active test of each catalyst in a cycle of 4 tests. Error bars are standard errors from the mean. Data set includes all catalyst batches; some tested at various stirring rates.

In another experiment, the immediate effect of stirring rate was evaluated. As expected, excessive H_2O_2 production in the medium quickly causes the deactivation of the enzyme; in Fig. S11, we confirm that increasing the stirring rate of the reactor briefly increases the activity

of the catalyst before the total deactivation of the enzyme. In this short time, the average PhEtOH production increased by 50 % from 1.5 mM/(g_{cat}.h) to 2.2 mM/(g_{cat}.h). This experiment highlights a possible resistance threshold of PaDa-I to H_2O_2 at about 1 mM.

To verify this value, an experiment was carried out to measure the direct effect of H2O2 concentration on the deactivation of the enzyme (Fig. S11B). Briefly, PaDa-I was diluted in various concentrations of H_2O_2 in water/acetone (2/3 v/v) medium for 15 minutes. Then, a mixture of 5-nitro-1,3-benzodioxole (NBD) and H_2O_2 was added to start the enzymatic reaction with 1 mM of H_2O_2 and excess NBD. This enables the observation of the effect of the incubation on PaDa-I without the effects of various H_2O_2 concentrations at the start of reaction: kinetics and theoretical yields of product are equal across all tests if no deactivation takes place. In this case, an obvious decrease in activity is observed even with the lowest concentration of H_2O_2 (0.25 mM). In accordance with the experiment in Fig. S11A, a massive drop in initial activity is observed at 1 mM H_2O_2, reaching 0 at 1.5 mM. With sufficient time, conversion of NBD is still observed above 1 mM, meaning that a small population of PaDa-I remains active, but this residual activity would not be sufficient to sustain a cascade reaction.

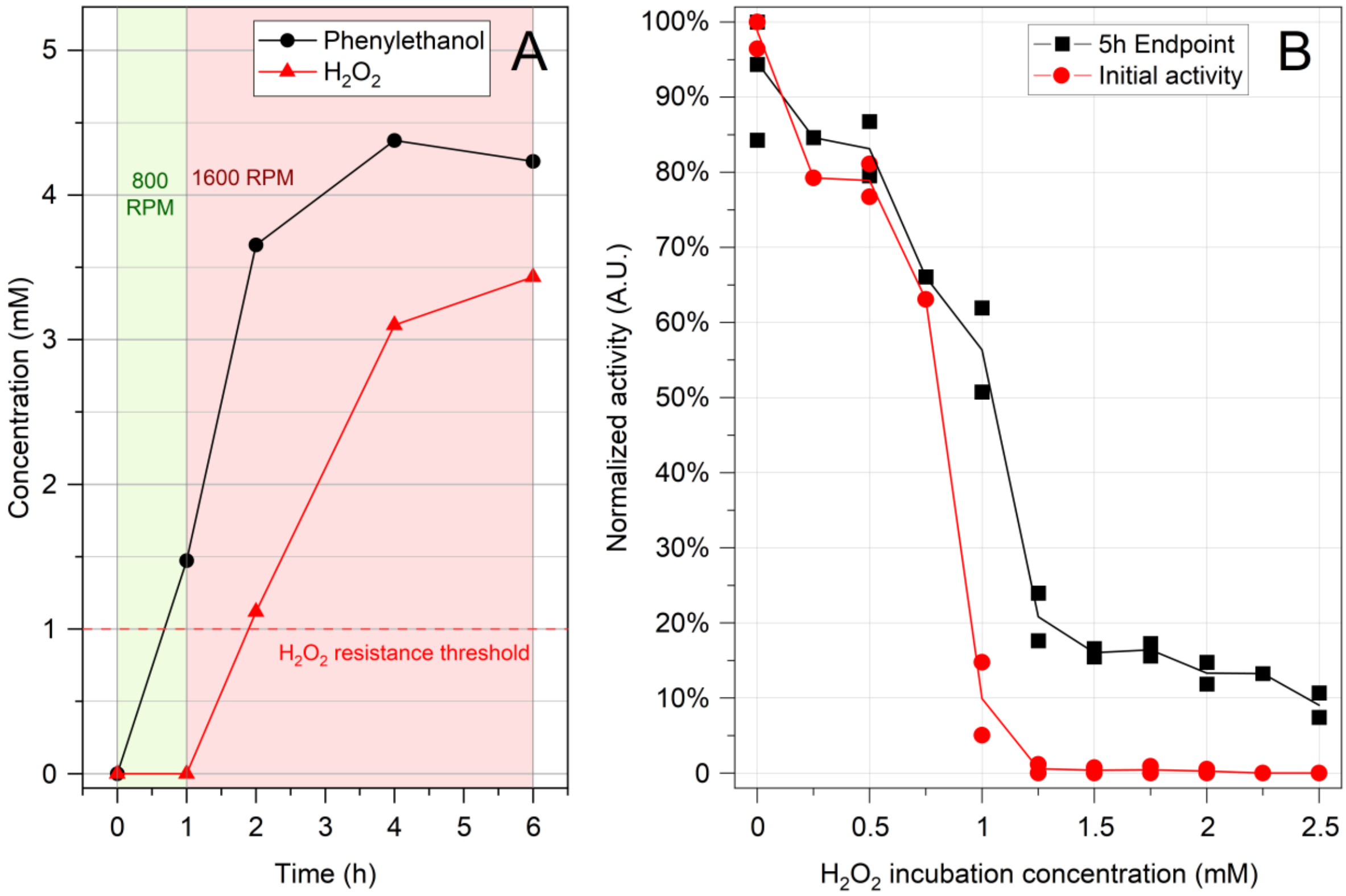

Fig. S11. Evaluation of the resistance threshold of PaDa-I towards H_2O_2. A: Cascade reaction kinetics of the hybrid catalysts. After 1 h, stir rate was increased from 800 to 1600 rpm. B: Enzymatic activity measured by NBD assay after 15 minutes of incubation in varying amounts of H_2O_2. Activities are normalized by the maximum measured value. Initial activity (red) is the slope of the absorbance kinetics curve measured for 30 seconds (during linear regime); black curve is measured from absorbance of the solution after 5 hours. Absorbances are corrected from a blank containing no enzyme.

S4: Insights on hybrid catalyst activity

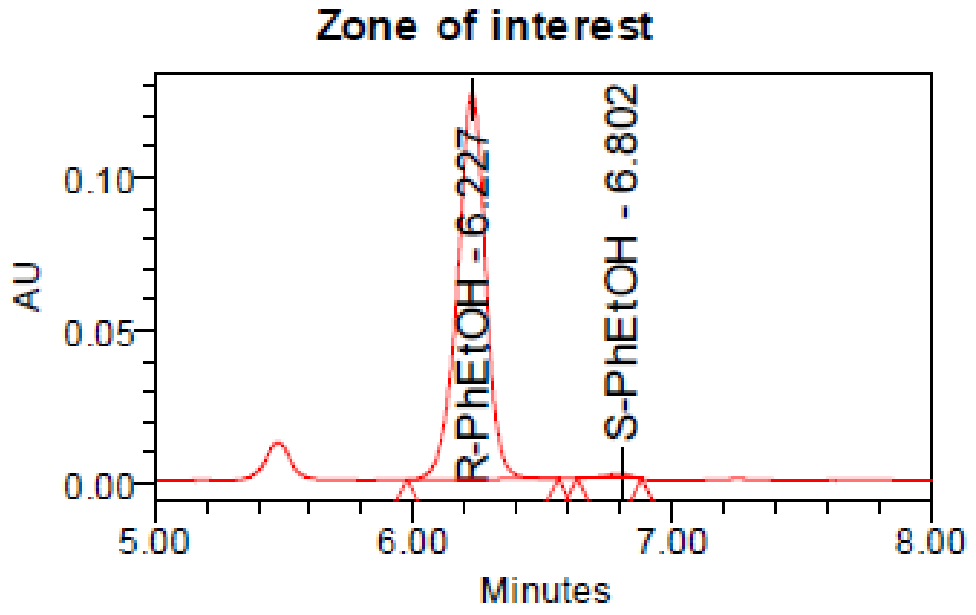

Peak Results

	Name	RT	Area	% Area
1	R-PhEtOH	6.227	951302	98.86
2	S-PhEtOH	6.802	11014	1.14

Fig. S12. Chromatogram and peak areas from chiral HPLC analysis

The value of *e.e.* is computed as $\frac{A_R - A_S}{A_R + A_S} \times 100$, with A_i the peak area of each enantiomer.

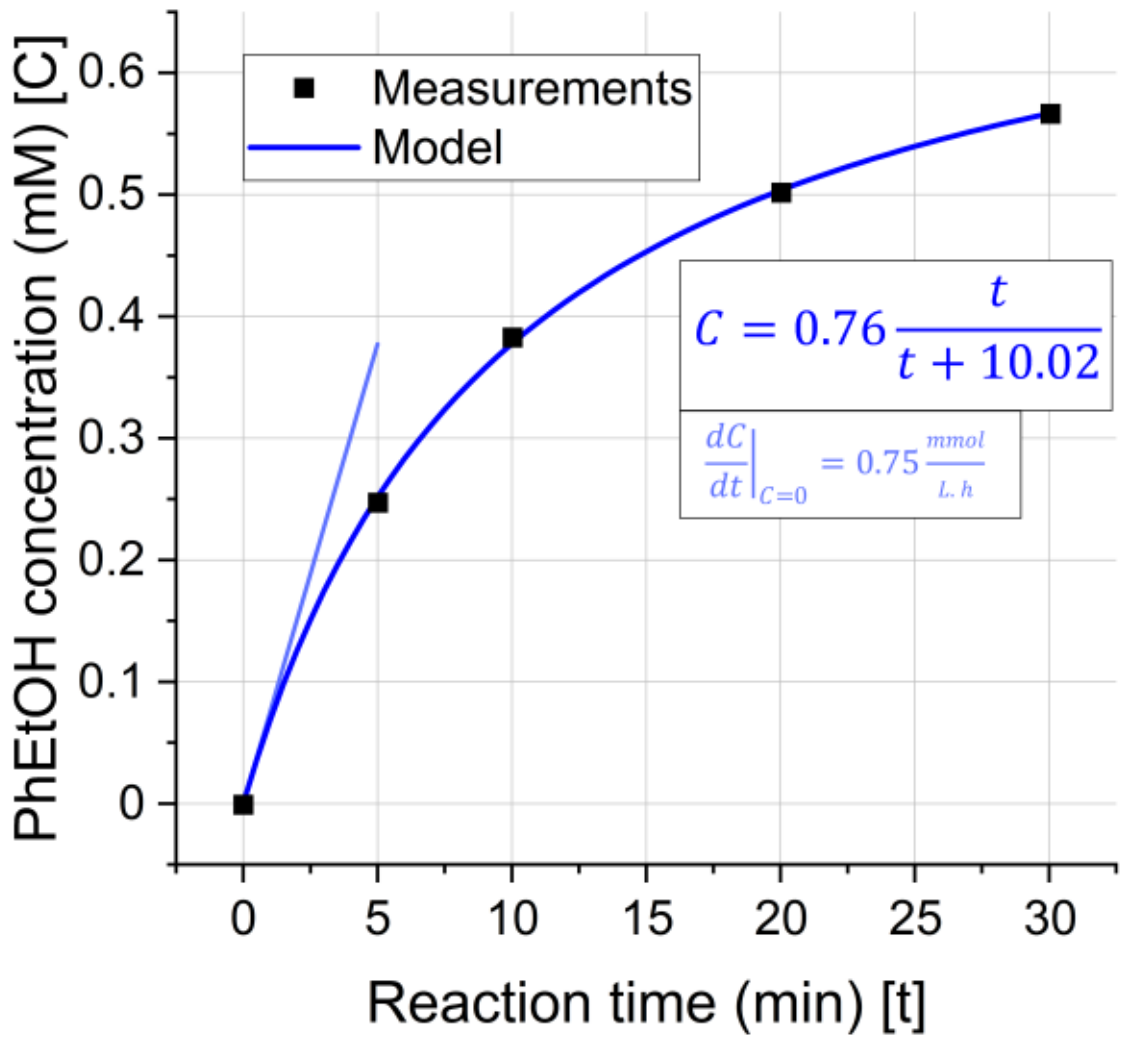

Fig. S13. time-course reaction of the oxidation of ethylbenzene to PhEtOH by EL-HCEHC (0.4 mg/mL) after addition of 1 mM H_2O_2.

Two experiments were conducted to measure the maximal turnover frequency of the hybrid catalyst (TOF). Briefly, EB oxidation was carried out in 48 wells microplates. Reaction medium containing EB, IST and the hybrid catalyst was prepared and dispensed in the wells. Reaction was started at the addition of H_2O_2 and carried out under orbital agitation of the microplate to keep the solid catalyst in suspension. One experiment measured the reaction kinetics over the course of 30 minutes (Fig. S13), the other compared the activities of the free enzyme and hybrid catalyst after 10 minutes of reaction, at various catalyst concentrations (Fig. S14). Each data point is taken from an independent well.

The concentration of PhEtOH produced by the hybrid catalyst in the span of 30 min in presence of 1 mM of exogenous H_2O_2 can be accurately approximated with the equation $C = 0.76 \frac{t}{t+10.02}$

(Fig. S13). From this equation, the theoretical initial productivity can be computed as 0.75 mmol.L^{-1}.min^{-1} or 11.3 mmol.g^{-1}.h^{-1}. To compare the activity of the hybrid catalyst and that of the free enzyme, similar tests were conducted, with varying concentrations of enzyme or hybrid catalyst (Fig. S14). Results show a decrease in activity of 59 % from the free enzyme to the hybrid catalyst. Though this value is high, it may be negatively impacted by two factors: an overestimation of the enzyme loading in the hybrid catalyst (see equation below) and/or a diffusion limitation of H_2O_2 towards the inside of the microspheres.

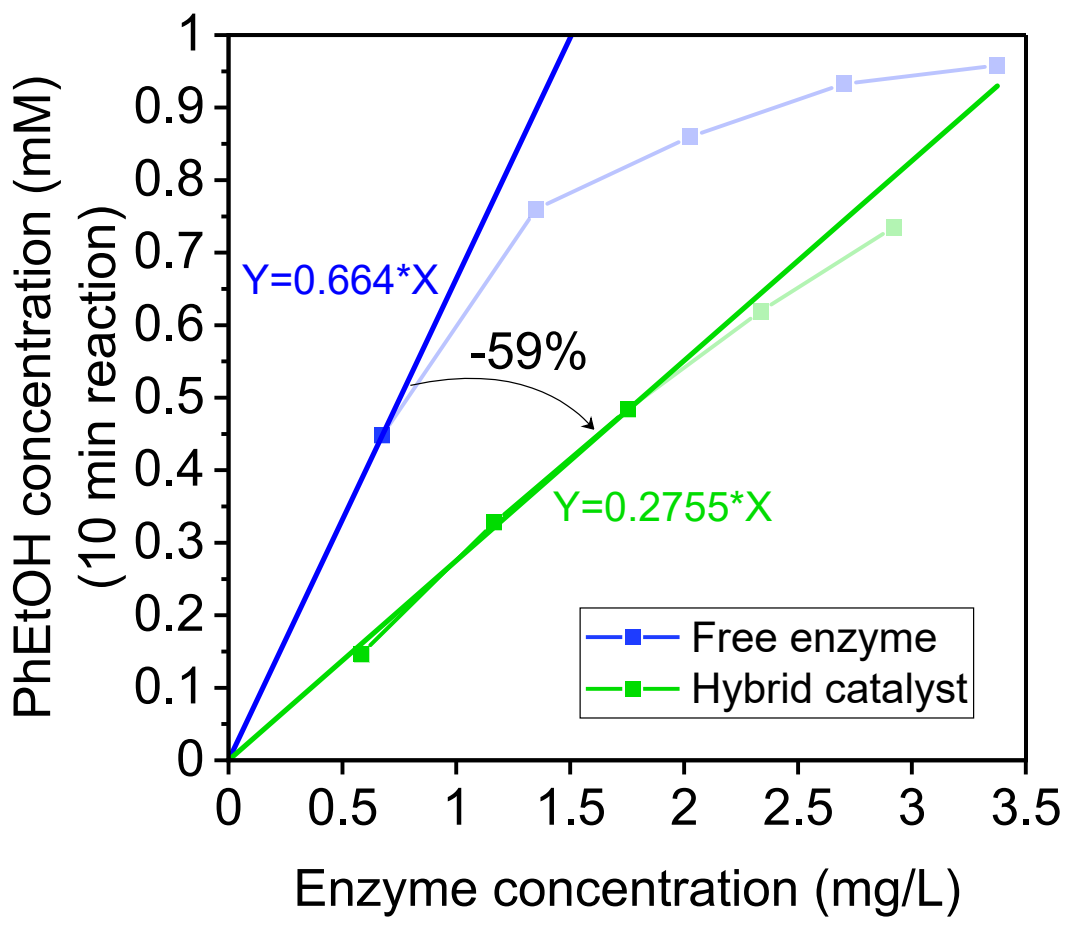

Fig. S14. comparison of the production of PhEtOH by free PaDa-I and the Hybrid catalyst in presence of 1 mM of H_2O_2 and various concentration of catalyst – normalized by concentration of enzyme. Enzyme concentration in the hybrid catalyst is taken as the theoretical maximum value. To account for the reduced activity measured at high conversion, values were compared using the linear region of the curves.

The concentration of enzyme in the hybrid catalyst was estimated as follows:

$$EL\left[{}^{\mu g_{enz}}/_{mg_{solid}}\right] = V_{pore}\left[{}^{cm^3}/_{mg_{solid}}\right] \times [Enz\ cc]\left[{}^{\mu g_{enz}}/_{cm^3}\right]$$

$$EL = 1.3\frac{cm^3}{mg_{solid}} \times 2.25\frac{\mu g_{enz}}{cm^3} = 2.925\frac{\mu g_{enz}}{mg_{solid}}$$

This estimation assumes that: 1) the entirety of the macroporosity (*i.e:* the microsphere cavity) of all the material is properly filled with the enzyme solution during preparation. 2) The concentration of the enzyme solution inside the sphere is equal to its concentration in the added solution. 3) 100% of the enzyme present inside the sphere is trapped. 5) No significant amount of enzyme remains outside of the material after thorough washing. 6) The mass of enzyme (>0.5 % w/w) does not significantly alter the mass of the catalyst, thus $m_{solid} = m_{hybr.cat.}$. These assumptions are backed by previous findings in our group, where theoretical enzyme loading was accurately confirmed by thermogravimetric analysis.[1]

These kinetic studies allow the computation of the maximum catalytic, normalized per mass of solid, or enzyme (Table S.1). The theoretical, maximum enzymatic turnover frequency (TOF) was computed as 3.9 $mmol.mg^{-1}.h^{-1}$ for the hybrid catalyst, and 4.0 for the free enzyme. This is coherent with the values of the non-encapsulated CLEAs, which retain most of the activity of the free enzyme. Here, we attribute the lower measured activity of the hybrid catalyst when H_2O_2 is added externally to diffusion limitation through the microsphere. As H_2O_2 is a small molecule compared to EB and PhEtOH, it can be assumed that these can also suffer from diffusion limitations during cascade reactions.

Table S2.: computed maximal activity of the hybrid catalyst

Variable	$mmol.g_{solid}^{-1}.h^{-1}$	$TOF\ (mmol.mg_{enz}^{-1}.h^{-1})$	$k_{cat}\ (mol_P.mol_{enz}^{-1}.s^{-1})$
Time	11.3	3.9	55
Enzyme conc.	4.8	1.7 (Free enz.: 4.0)	23

The proximity effect of combining PaDa-I and AuPd/TS-1 catalyst together on the same solid was evaluated by performing the reaction using entrapped PaDa-I in metal-free TS-1 spheres (1 g/L). The supply of H_2O_2 was assured by the addition of 1 g/L of SD-AuPD/TS-1 to match the H_2O_2 production of a hybrid catalyst (Fig. S15). The two-catalysts mixture performs initially well, but H_2O_2 immediately accumulates in the reaction medium; after 2 hours of reaction, H_2O_2 visibly starts degrading the enzyme. After 4 hours, the enzyme is completely deactivated. This shows a clear benefit to the survival of the enzyme of having the two catalysts on the same solid. Here, this effect is attributed to a potential effect of the peroxidase activity of PaDa-I: it is likely that any excess H_2O_2 produced in close proximity of the enzyme is regulated by its natural protection mechanism. When the catalysts are physically segregated, H_2O_2 can uncontrollably accumulate outside of the microspheres before the enzymes have time to react, causing their eventual deactivation.

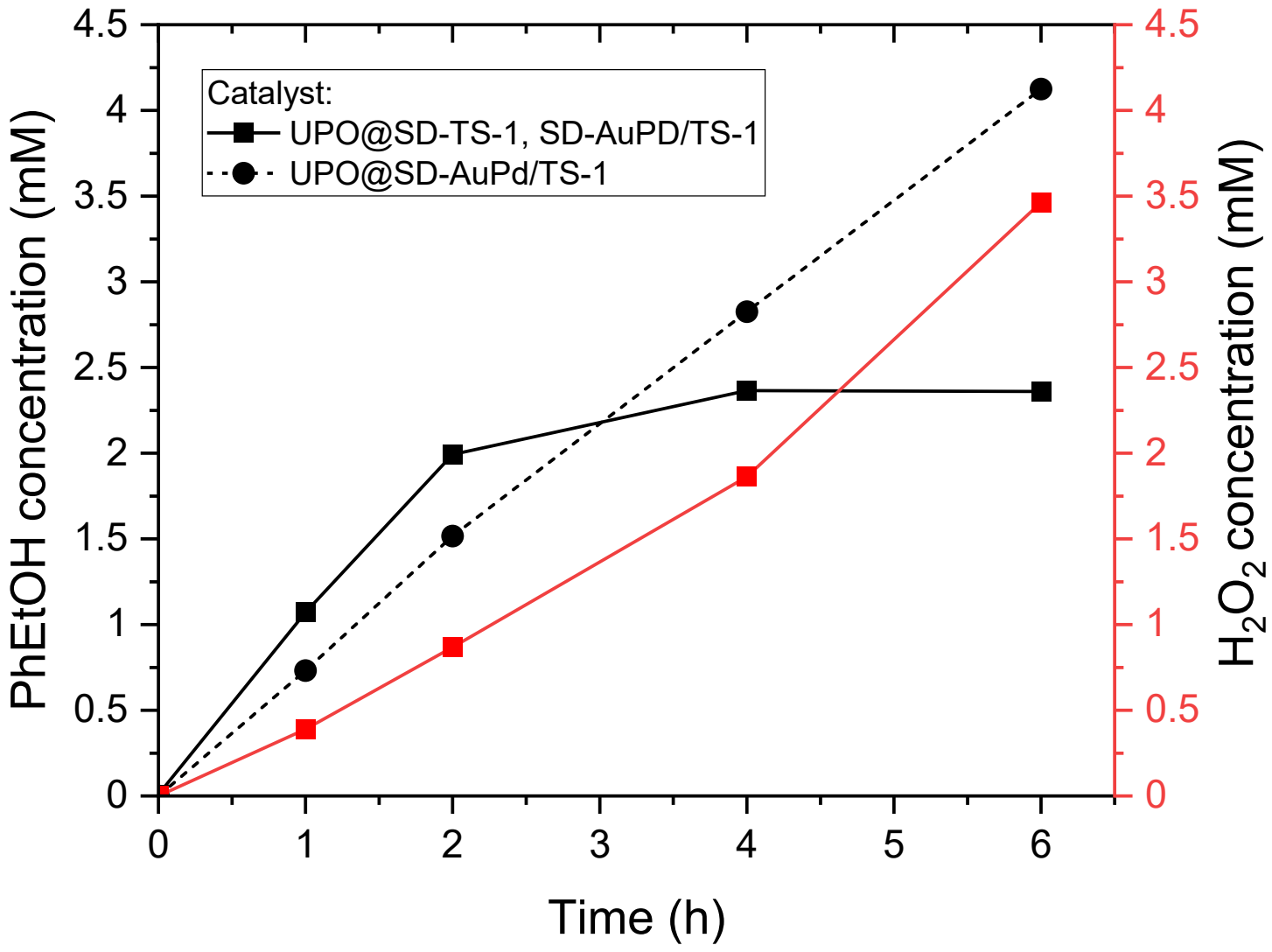

Fig. S15. Catalytic activity of immobilized UPO on metal-free TS-1 microspheres (SD-TS-1) with SD-AuPd/TS-1 as H_2O_2 generation catalyst. Hybrid catalyst performance (dotted line) added for comparison

S5: Enzyme production

pPICZB-AaeUPO*-CHis6 : genetic sequence

AGATCTAACATCCAAAGACGAAAGGTTGAATGAAACCTTTTTGCCATCCGACATCC
ACAGGTCCATTCTCACACATAAGTGCCAAACGCAACAGGAGGGGATACACTAGCA
GCAGACCGTTGCAAACGCAGGACCTCCACTCCTCTTCTCCTCAACACCCACTTTTG
CCATCGAAAAACCAGCCCAGTTATTGGGCTTGATTGGAGCTCGCTCATTCCAATTC
CTTCTATTAGGCTACTAACACCATGACTTTATTAGCCTGTCTATCCTGGCCCCCCTGG
CGAGGTTCATGTTTGTTTATTTCCGAATGCAACAAGCTCCGCATTACACCCGAACAT
CACTCCAGATGAGGGCTTTCTGAGTGTGGGGTCAAATAGTTTCATGTTCCCCAAAT
GGCCCAAAACTGACAGTTTAAACGCTGTCTTGGAACCTAATATGACAAAAGCGTG
ATCTCATCCAAGATGAACTAAGTTTGGTTCGTTGAAATGCTAACGGCCAGTTGGTC
AAAAAGAAACTTCCAAAAGTCGGCATACCGTTTGTCTTGTTTGGTATTGATTGACG
AATGCTCAAAAATAATCTCATTAATGCTTAGCGCAGTCTCTCTATCGCTTCTGAACC
CCGGTGCACCTGTGCCGAAACGCAAATGGGGAAACACCCGCTTTTTGGATGATTAT
GCATTGTCTCCACATTGTATGCTTCCAAGATTCTGGTGGGAATACTGCTGATAGCCT
AACGTTCATGATCAAAATTTAACTGTTCTAACCCCTACTTGACAGCAATATATAAAC
AGAAGGAAGCTGCCCTGTCTTAAACCTTTTTTTTTATCATCATTATTAGCTTACTTTC
ATAATTGCGACTGGTTCCAATTGACAAGCTTTTGATTTTAACGACTTTTAACGACAA
CTTGAGAAGATCAAAAAACAACTAATTATTCGAAACGAGGAATTCATGAAATATTT
TCCCCTGTTCCCAACCTTGGTCTATGCAGTTGGTGTCGTTGCTTTTCCTGATTACGC
CTCATTGGCCGGCCTCAGCCAGCAGGAATTGGACGCTATAATCCCAACACTCGAGG
CCCGAGAGCCAGGATTACCTCCTGGTCCTCTCGAGAATAGCTCTGCAAAGTTGGTG
AACGACGAGGCTCACCCATGGAAGCCGCTTCGACCTGGCGATATTCGTGGACCTT
GCCCTGGTTTGAATACTCTGGCATCTCACGGGTACCTCCCGAGAAATGGCGTTGCA
ACCCCGGCTCAAATAATAAACGCGGTTCAGGAAGGATTTAATTTCGACAATCAAGC
CGCAATTTTCGCCACATATGCGGCCCACCTTGTGGACGGCAATCTCATTACGGACTT

GCTGAGCATCGGACGCAAGACGCGGCTCACTGGGCCTGATCCACCACCCCCCGCT
TCCGTTGGTGGACTCAATGAGCATGGCACCTTCGAAGGCGACGCCAGTATGACCC
GAGGTGACGCATTCTTTGGCAACAACCACGATTTCAATGAGACGCTCTTCGAACA
GTTGGTTGACTACAGCAACCGATTTGGAGGAGGAAAATACAATCTTACCGTCGCG
GGGGAGCTCCGTTTCAAGCGCATTCAAGACTCCATTGCGACCAACCCCAATTTCTC
CTTTGTTGACTTTAGGTTCTTTACTGCTTACGGCGAAACCACCTTCCCCGCGAATCT
TTTTGTGGATGGGCGCAGGGACGACGGCCAGCTAGATATGGATGCTGCACGGAGT
TTTTTCCAATTCAGCCGTATGCCTGACGATTTCTTCCGCGCACCCAGCCCGAGAAG
TGGCACAGGAGTCGAGGTAGTTGTACAGGCTCATCCTATGCAGCCCGGAAGAAAT
GTCGGCAAGATCAACAGCTACACCGTCGACCCAACATCCTCTGACTTTTCCACCCC
CTGCTTGATGTACGAGAAATTCGTCAACATAACGGTCAAGTCACTCTACCCGAATC
CGACGGTGCAGCTTCGCAAAGCCCTTAATACGAATCTCGATTTCTTGTTCCAGGGA
GTCGCCGCTGGATGTACCCAGGTTTCCCATACGGGCGAGATGGTCATCATCATCA
CCACCACTGATCTAGAACAAAAACTCATCTCAGAAGAGGATCTGAATAGCGCCGT
CGACCATCATCATCATCATCATTGAGTTTGTAGCCTTAGACATGACTGTTCCTCAGT
TCAAGTTGGGCACTTACGAGAAGACCGGTCTTGCTAGATTCTAATCAAGAGGATGT
CAGAATGCCATTTGCCTGAGAGATGCAGGCTTCATTTTTGATACTTTTTTATTTGTA
ACCTATATAGTATAGGATTTTTTTTGTCATTTTGTTTCTTCTCGTACGAGCTTGCTCCT
GATCAGCCTATCTCGCAGCTGATGAATATCTTGTGGTAGGGGTTTGGGAAAATCATT
CGAGTTTGATGTTTTTCTTGGTATTTCCCACTCCTCTTCAGAGTACAGAAGATTAAG
TGAGACCTTCGTTTGTGCGGATCCCCCACACACCATAGCTTCAAAATGTTTCTACTC
CTTTTTTACTCTTCCAGATTTTCTCGGACTCCGCGCATCGCCGTACCACTTCAAAAC
ACCCAAGCACAGCATACTAAATTTCCCCTCTTTCTTCCTCTAGGGTGTCGTTAATTA
CCCGTACTAAAGGTTTGGAAAAGAAAAAAGAGACCGCCTCGTTTCTTTTTCTTCGT
CGAAAAAGGCAATAAAAATTTTTATCACGTTTCTTTTTCTTGAAATTTTTTTTTTTAG
TTTTTTTCTCTTTCAGTGACCTCCATTGATATTTAAGTTAATAAACGGTCTTCAATTT
CTCAAGTTTCAGTTTCATTTTTCTTGTTCTATTACAACTTTTTTTACTTCTTGTTCATT
AGAAAGAAAGCATAGCAATCTAATCTAAGGGGCGGTGTTGACAATTAATCATCGGC
ATAGTATATCGGCATAGTATAATACGACAAGGTGAGGAACTAAACCATGGCCAAGT
TGACCAGTGCCGTTCCGGTGCTCACCGCGCGCGACGTCGCCGGAGCGGTCGAGTT
CTGGACCGACCGGCTCGGGTTCTCCCGGGACTTCGTGGAGGACGACTTCGCCGGT
GTGGTCCGGGACGACGTGACCCTGTTCATCAGCGCGGTCCAGGACCAGGTGGTGC
CGGACAACACCCTGGCCTGGGTGTGGGTGCGCGGCCTGGACGAGCTGTACGCCG
AGTGGTCGGAGGTCGTGTCCACGAACTTCCGGGACGCCTCCGGGCCGGCCATGAC
CGAGATCGGCGAGCAGCCGTGGGGGCGGGAGTTCGCCCTGCGCGACCCGGCCGG
CAACTGCGTGCACTTCGTGGCCGAGGAGCAGGACTGACACGTCCGACGGCGGCC
CACGGGTCCCAGGCCTCGGAGATCCGTCCCCCTTTTCCTTTGTCGATATCATGTAAT
TAGTTATGTCACGCTTACATTCACGCCCTCCCCCCACATCCGCTCTAACCGAAAAG
GAAGGAGTTAGACAACCTGAAGTCTAGGTCCCTATTTATTTTTTTATAGTTATGTTA
GTATTAAGAACGTTATTTATATTTCAAATTTTTCTTTTTTTTCTGTACAGACGCGTGT
ACGCATGTAACATTATACTGAAAACCTTGCTTGAGAAGGTTTTGGGACGCTCGAAG
GCTTTAATTTGCAAGCTGGAGACCAACATGTGAGCAAAAGGCCAGCAAAAGGCCA
GGAACCGTAAAAAGGCCGCGTTGCTGGCGTTTTTCCATAGGCTCCGCCCCCCTGAC
GAGCATCACAAAAATCGACGCTCAAGTCAGAGGTGGCGAAACCCGACAGGACTAT
AAAGATACCAGGCGTTTCCCCCTGGAAGCTCCCTCGTGCGCTCTCCTGTTCCGACC
CTGCCGCTTACCGGATACCTGTCCGCCTTTCTCCCTTCGGGAAGCGTGGCGCTTTC
TCAATGCTCACGCTGTAGGTATCTCAGTTCGGTGTAGGTCGTTCGCTCCAAGCTGG

GCTGTGTGCACGAACCCCCCGTTCAGCCCGACCGCTGCGCCTTATCCGGTAACTAT
CGTCTTGAGTCCAACCCGGTAAGACACGACTTATCGCCACTGGCAGCAGCCACTG
GTAACAGGATTAGCAGAGCGAGGTATGTAGGCGGTGCTACAGAGTTCTTGAAGTG
GTGGCCTAACTACGGCTACACTAGAAGGACAGTATTTGGTATCTGCGCTCTGCTGA
AGCCAGTTACCTTCGGAAAAAGAGTTGGTAGCTCTTGATCCGGCAAACAAACCAC
CGCTGGTAGCGGTGGTTTTTTTGTTTGCAAGCAGCAGATTACGCGCAGAAAAAAA
GGATCTCAAGAAGATCCTTTGATCTTTTCTACGGGGTCTGACGCTCAGTGGAACGA
AAACTCACGTTAAGGGATTTGGTCATGAGATC

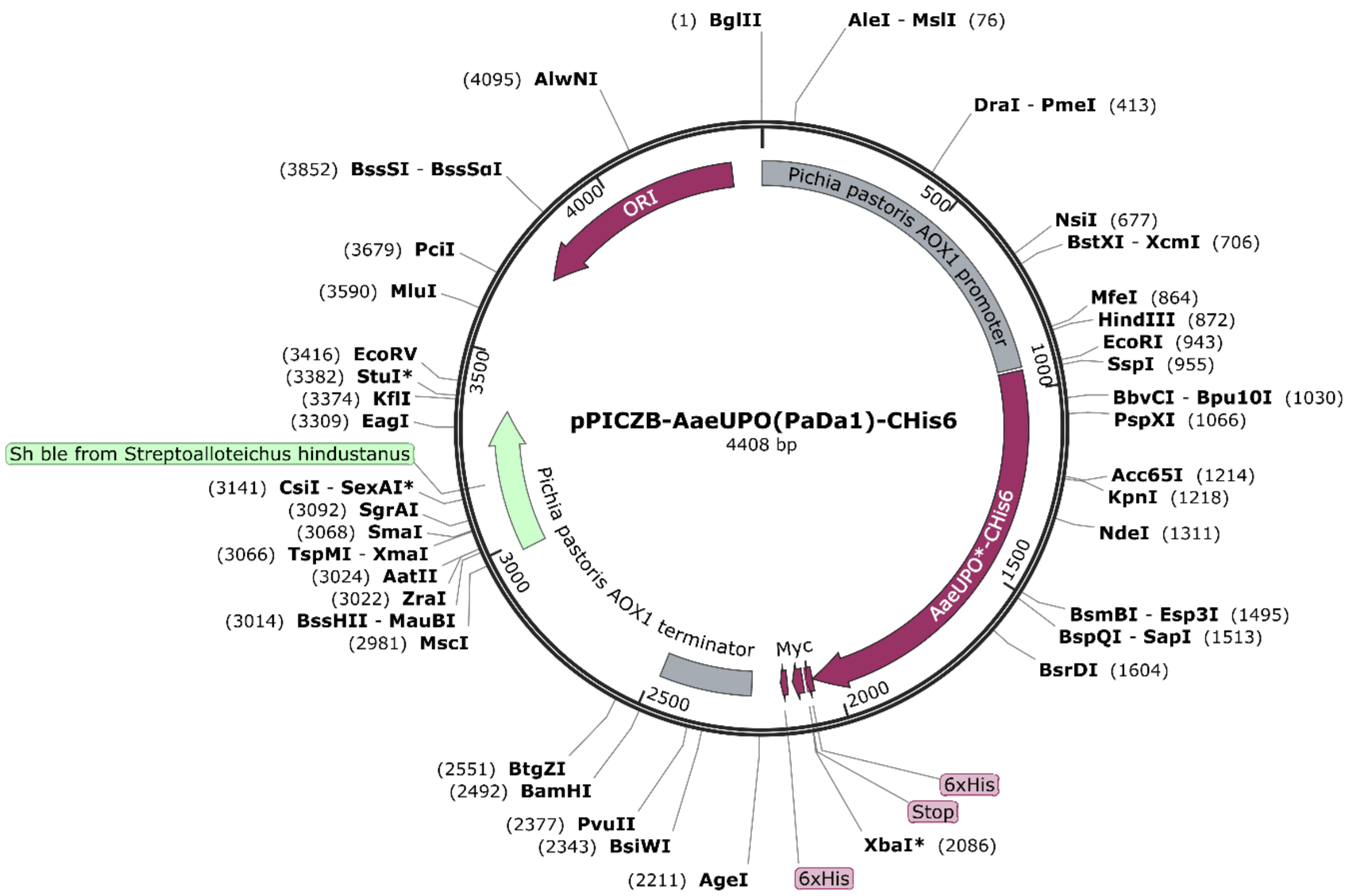

Fig. S16: plasmid map

Protein sequence :

MKYFPLFPTLVYAVGVVAFPDYASLAGLSQQELDAIIPTLEAREPGLPPGPLENSSAKL
VNDEAHPWKPLRPGDIRGPCPGLNTLASHGYLPRNGVATPAQIINAVQEGFNFDNQAA
IFATYAAHLVDGNLITDLLSIGRKTRLTGPDPPPPASVGGLNEHGTFEGDASMTRGDAF
FGNNHDFNETLFEQLVDYSNRFGGGKYNLTVAGELRFKRIQDSIATNPNFSFVDFRFFT
AYGETTFPANLFVDGRRDDGQLDMDAARSFFQFSRMPDDFFRAPSPRSGTGVEVVVVQ
AHPMQPGRNVGKINSYTVDPTSSDFSTPCLMYEKFVNITVKSLYPNPTVQLRKALNTN
LDFLFQGVAAGCTQVFPYGRDGHHHHHH

SDS-PAGE

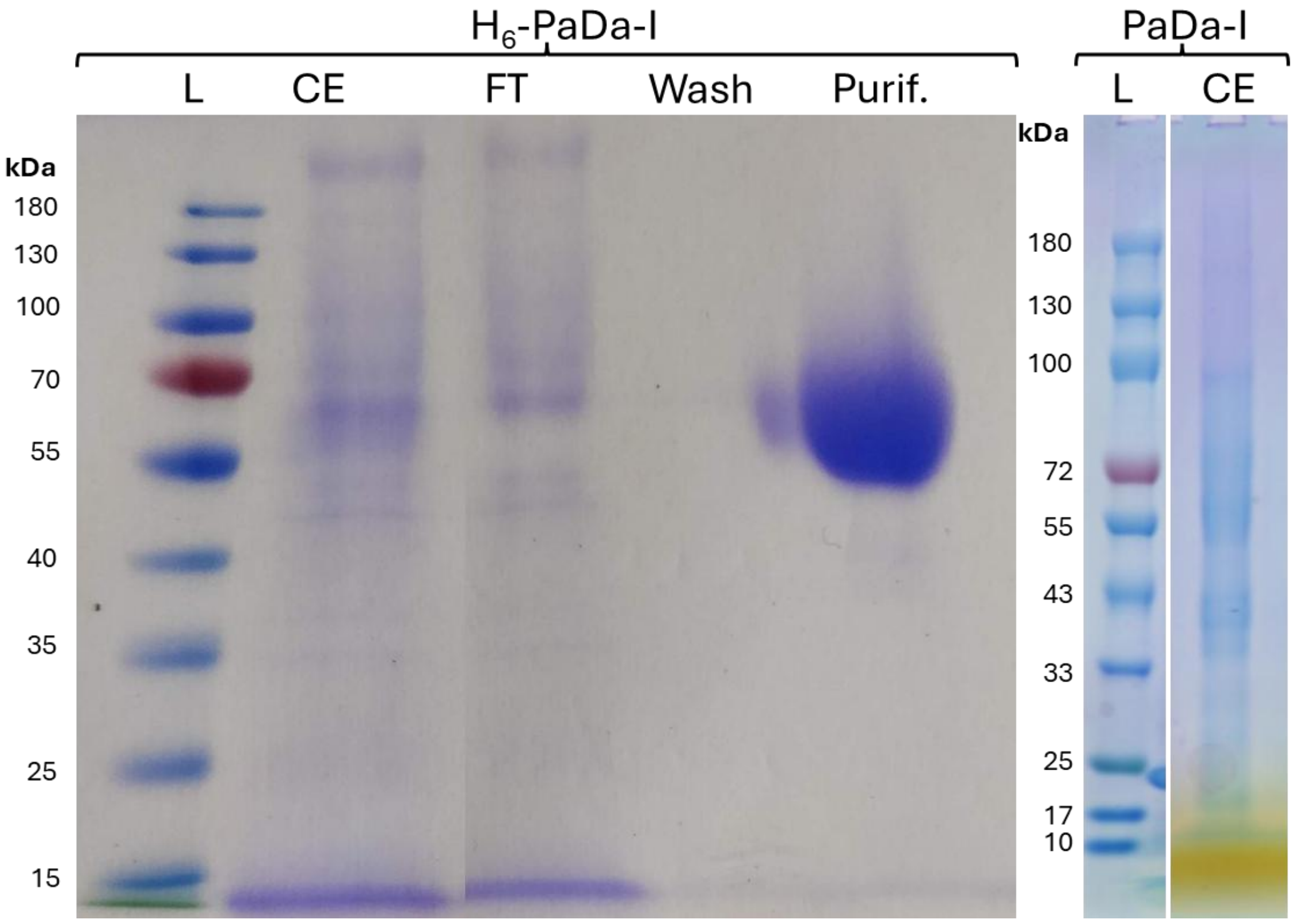

Fig. S17. SDS-Page of H_6-PaDa-I and PaDa-I. L: ladder, CE: crude extract, FT : flow-through of purification procedure, W: column wash, Purif: purified protein.

S6: Discussion on safety and green metrics

The claim for greenness of the proposed catalyst relies primarily on the removal of commercially produced H_2O_2 for the system. Indeed, hydrogen peroxide, though cheap and easy to handle, is industrially produced using the anthraquinone (AO) process. The latter uses similar catalysts (Pd on C, Al_2O_3, SiO_2, or $Si_{1-x}Al_xO_2$)[17–19], so the footprint related to the catalyst manufacture is only displaced. Moreover, AO is energy intensive because it is operated at high P, T, and requires extensive purification. Its energy footprint is estimated at 2.9-5.7 kJ/kg_{H2O2}, excluding H_2 production.[18] It is also a process that is plagued by important VOC emissions and solvent use.[20] Though well-mastered and controlled industrially, it operates with H_2 at 60-90%.[19] The process described here (initially proposed by the Hutchings group[12]) is solely fueled by H_2 and O_2 gases, which can be highly diluted, mitigating EHS risks. As the system was only tested at the laboratory scale (25 mL) and as we focused on catalytic activity and stability, no definitive E-factor can be computed. However, comparatively to a similar process working with free UPO and external addition of commercial H_2O_2, the following differences arise:

- no prior H_2O_2 production – removal of abovementioned Scope 2 energy use and VOC pollution,

- no H_2O_2 pump (replaced with gas tanks) – reduction in moving parts,
- lower risk of H_2O_2 poisoning of the enzyme from uneven mixing – common risk at high scale[21,22],
- use of a fully heterogeneous catalyst.

The latter point is crucial, as it enables the use and reuse of enzymes in multiple batches or potentially the development of continuous processes. Thus, the chemo-enzymatic system ensures a more sustainable use of the enzyme. To show the gains in stability, fed-batch experiments were performed to evaluate the impact of gradual H_2O_2 addition on enzymatic activity. Fig. S18 shows that, as feeding rate increases, the enzyme starts losing activity – even at feeding rates of 20,000 h^{-1}. On the contrary, the enzymatic activity was sustained under in cascade conditions where the in situ H_2O_2 production rate corresponds to roughly 38,000 h^{-1}.

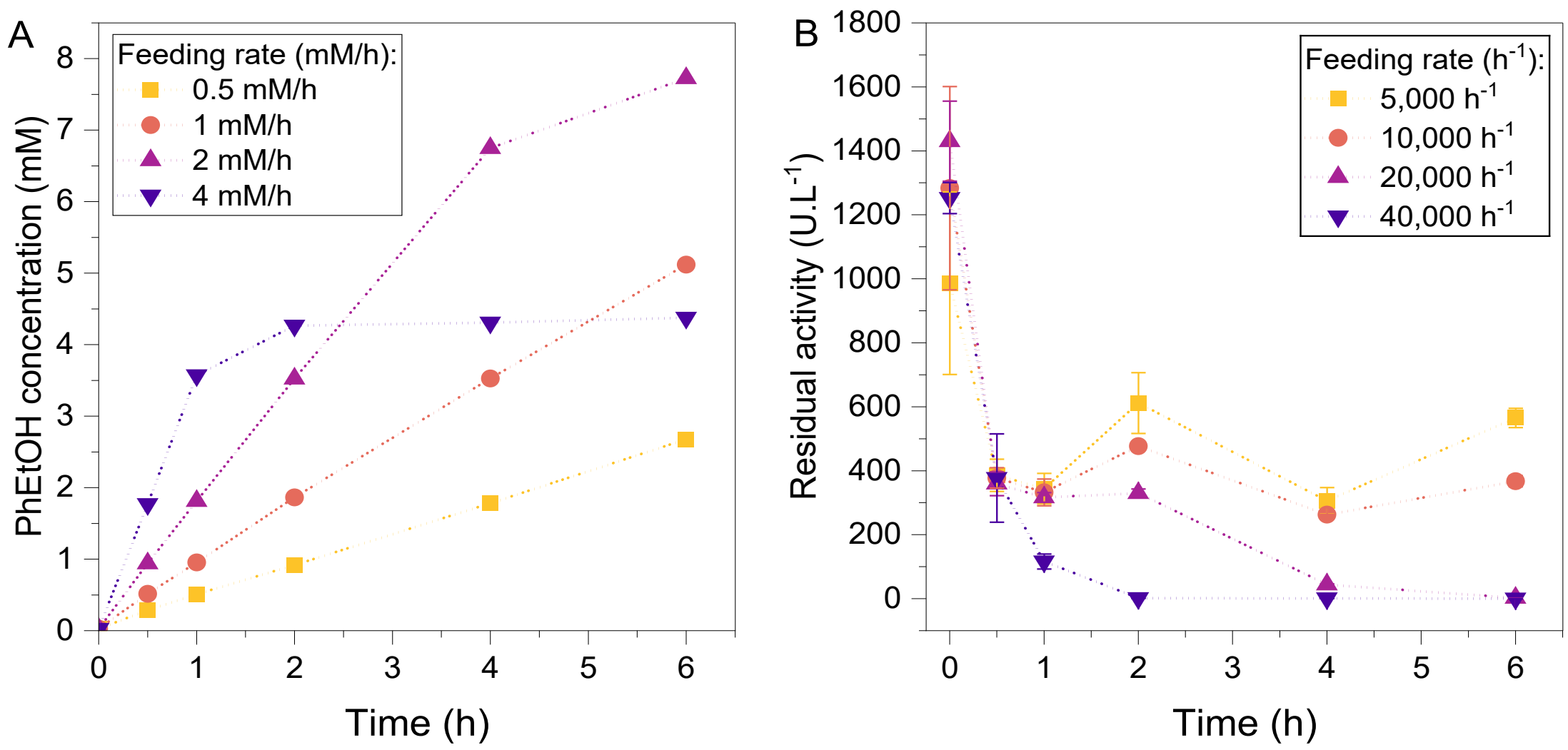

Fig. S18. Fed-batch experiments using H_6-PaDa-I in Tris-HCl (pH 8.5). A: PhEtOH production, B: Residual activity measured by ABTS assay in the reaction medium diluted 60 times in a solution containing citrate buffer (pH 4.1), ABTS 0.5 mM and H_2O_2 1 mM. Feeding rates correspond to the amount of H_2O_2 provided to the fed-batch system (expressed either in mM/h or in h^{-1}).

References:

(1) Smeets, V.; Baaziz, W.; Ersen, O.; Gaigneaux, E. M.; Boissière, C.; Sanchez, C.; Debecker, D. P. Hollow Zeolite Microspheres as a Nest for Enzymes: A New Route to Hybrid Heterogeneous Catalysts. *Chem. Sci.* **2020**, *11* (4), 954–961. https://doi.org/10.1039/C9SC04615A.

(2) Van der Verren, M.; Smeets, V.; vander Straeten, A.; Dupont-Gillain, C.; Debecker, D. P. Hybrid Chemoenzymatic Heterogeneous Catalyst Prepared in One Step from Zeolite

Nanocrystals and Enzyme–Polyelectrolyte Complexes. *Nanoscale Adv.* **2021**, *3* (6), 1646–1655. https://doi.org/10.1039/D0NA00834F.
(3) Thangaraj, A.; Eapen, M. J.; Sivasanker, S.; Ratnasamy, P. Studies on the Synthesis of Titanium Silicalite, TS-1. *Zeolites* **1992**, *12* (8), 943–950. https://doi.org/10.1016/0144-2449(92)90159-M.
(4) Sankar, M.; He, Q.; Morad, M.; Pritchard, J.; Freakley, S. J.; Edwards, J. K.; Taylor, S. H.; Morgan, D. J.; Carley, A. F.; Knight, D. W.; Kiely, C. J.; Hutchings, G. J. Synthesis of Stable Ligand-Free Gold–Palladium Nanoparticles Using a Simple Excess Anion Method. *ACS Nano* **2012**, *6* (8), 6600–6613. https://doi.org/10.1021/nn302299e.
(5) Freakley, S. J.; Kochius, S.; van Marwijk, J.; Fenner, C.; Lewis, R. J.; Baldenius, K.; Marais, S. S.; Opperman, D. J.; Harrison, S. T. L.; Alcalde, M.; Smit, M. S.; Hutchings, G. J. A Chemo-Enzymatic Oxidation Cascade to Activate C–H Bonds with in Situ Generated H2O2. *Nat Commun* **2019**, *10* (1), 4178. https://doi.org/10.1038/s41467-019-12120-w.
(6) Pritchard, J. C.; He, Q.; Ntainjua, E. N.; Piccinini, M.; Edwards, J. K.; Herzing, A. A.; Carley, A. F.; Moulijn, J. A.; Kiely, C. J.; Hutchings, G. J. The Effect of Catalyst Preparation Method on the Performance of Supported Au–Pd Catalysts for the Direct Synthesis of Hydrogen Peroxide. *Green Chem.* **2010**, *12* (5), 915–921. https://doi.org/10.1039/B924472G.
(7) Molina-Espeja, P.; Garcia-Ruiz, E.; Gonzalez-Perez, D.; Ullrich, R.; Hofrichter, M.; Alcalde, M. Directed Evolution of Unspecific Peroxygenase from Agrocybe Aegerita. *Applied and Environmental Microbiology* **2014**, *80* (11), 3496–3507. https://doi.org/10.1128/AEM.00490-14.
(8) Molina-Espeja, P.; Ma, S.; Mate, D. M.; Ludwig, R.; Alcalde, M. Tandem-Yeast Expression System for Engineering and Producing Unspecific Peroxygenase. *Enzyme and Microbial Technology* **2015**, *73–74*, 29–33. https://doi.org/10.1016/j.enzmictec.2015.03.004.
(9) Introvigen. Pichia Fermentation Process Guidelines, 2010. https://documents.thermofisher.com/TFS-Assets/LSG/manuals/pichiaferm_prot.pdf.
(10) Yang, Y.; Schrand, N.; Sha, M. A Guide to Pichia Pastoris Fermentation in Single-Use Stirred-Tank Bioreactors. *Eppendorf 38*.
(11) Nintzel, F. E. H.; Wu, Y.; Planchestainer, M.; Held, M.; Alcalde, M.; Hollmann, F. An Alginate-Confined Peroxygenase-CLEA for Styrene Epoxidation. *Chem. Commun.* **2021**, *57* (47), 5766–5769. https://doi.org/10.1039/D1CC01868J.
(12) Edwards, J. K.; Solsona, B.; N, E. N.; Carley, A. F.; Herzing, A. A.; Kiely, C. J.; Hutchings, G. J. Switching Off Hydrogen Peroxide Hydrogenation in the Direct Synthesis Process. *Science* **2009**, *323* (5917), 1037–1041. https://doi.org/10.1126/science.1168980.
(13) Santos, A.; Lewis, R. J.; Malta, G.; Howe, A. G. R.; Morgan, D. J.; Hampton, E.; Gaskin, P.; Hutchings, G. J. Direct Synthesis of Hydrogen Peroxide over Au–Pd Supported Nanoparticles under Ambient Conditions. *Ind. Eng. Chem. Res.* **2019**, *58* (28), 12623–12631. https://doi.org/10.1021/acs.iecr.9b02211.
(14) Brehm, J.; Lewis, R. J.; Richards, T.; Qin, T.; Morgan, D. J.; Davies, T. E.; Chen, L.; Liu, X.; Hutchings, G. J. Enhancing the Chemo-Enzymatic One-Pot Oxidation of Cyclohexane via In Situ H2O2 Production over Supported Pd-Based Catalysts. *ACS Catal.* **2022**, *12* (19), 11776–11789. https://doi.org/10.1021/acscatal.2c03051.
(15) Genc, A.; Marlowe, J.; Jalil, A.; Belzberg, D.; Kovarik, L.; Christopher, P. A Versatile Machine Learning Workflow for High-Throughput Analysis of Supported Metal Catalyst Particles. *Ultramicroscopy* **2025**, *271*, 114116. https://doi.org/10.1016/j.ultramic.2025.114116.

(16) Velasco-Lozano, S. Immobilization of Enzymes as Cross-Linked Enzyme Aggregates: General Strategy to Obtain Robust Biocatalysts. In *Immobilization of Enzymes and Cells: Methods and Protocols*; Guisan, J. M., Bolivar, J. M., López-Gallego, F., Rocha-Martín, J., Eds.; Springer US: New York, NY, 2020; pp 345–361. https://doi.org/10.1007/978-1-0716-0215-7_23.
(17) Goor, G.; Glenneberg, J.; Jacobi, S.; Dadabhoy, J.; Candido, E. Hydrogen Peroxide. In *Ullmann's Encyclopedia of Industrial Chemistry*; Wiley, 2019; pp 1–40. https://doi.org/10.1002/14356007.a13_443.pub3.
(18) Theofanidis, S.-A.; Georgiadis, A. G.; Hop, C. J. W.; Yu, X.; Yfanti, V.-L.; Fayet, G.; Villemur, C.; Vleeming, H.; Delikonstantis, E.; Heyn, R. H. Alternative H2O2 Production Processes: An Outlook on Candidate Technologies Beyond the Anthraquinone Process. *ACS Omega* **2025**, *10* (50), 61076–61095. https://doi.org/10.1021/acsomega.5c07503.
(19) Vandenbussche, A.; Dhaese, P. M.; Bloomfield, S.; Janssens, F. Process for Producing Hydrogen Peroxide. EP2766300B1, May 20, 2020. https://patents.google.com/patent/EP2766300B1/en%C2%A0 (accessed 2026-06-16).
(20) Jia, Y.; Bai, Y.; Chang, J.; Zhai, Y.; Zhang, T.; Ren, K.; Hong, J. Life Cycle Assessment of Hydrogen Peroxide Produced from Mainstream Hydrogen Sources in China. *Journal of Cleaner Production* **2022**, *352*, 131655. https://doi.org/10.1016/j.jclepro.2022.131655.
(21) Prayag, K. S.; Jindal, A. B. Scale-Up of Liquid Mixing Process. In *Pharmaceutical Process Engineering and Scale-up Principles*; Jindal, A. B., Ed.; Springer Nature Switzerland: Cham, 2023; pp 131–148. https://doi.org/10.1007/978-3-031-31380-6_10.
(22) Rosseburg, A.; Fitschen, J.; Wutz, J.; Wucherpfennig, T.; Schlüter, M. Hydrodynamic Inhomogeneities in Large Scale Stirred Tanks – Influence on Mixing Time. *Chemical Engineering Science* **2018**, *188*, 208–220. https://doi.org/10.1016/j.ces.2018.05.008.
